# Glycosaminoglycans activate peptidylarginine deiminase 4 by enhancing calcium affinity

**DOI:** 10.1073/pnas.2508369122

**Published:** 2025-10-30

**Authors:** Grzegorz P. Bereta, Ewa Bielecka, Karolina Marzec, Łukasz Pijanowski, Artur P. Biela, Piotr Wilk, Marta Kamińska, Jakub Nowak, Elżbieta Wątor-Wilk, Przemysław Grudnik, Dominik Kowalczyk, Joanna Kozieł, Piotr Mydel, Marcin Poręba, Tomasz Kantyka

**Affiliations:** ^a^Malopolska Centre of Biotechnology, Jagiellonian University, Krakow 30-387, Poland; ^b^Department of Microbiology, Faculty of Biochemistry, Biophysics and Biotechnology, Jagiellonian University, Krakow 30-387, Poland; ^c^SOLARIS National Synchrotron Radiation Centre, Jagiellonian University, Krakow 30-392, Poland; ^d^Broegelmann Research Laboratory, University of Bergen, Bergen NO-5020, Norway; ^e^Department of Chemical Biology and Bioimaging, Faculty of Chemistry, Wroclaw University of Science and Technology, Wroclaw 50-372, Poland

**Keywords:** citrullination, rheumatoid arthritis, glycosaminoglycan, peptidylarginine deiminase, PAD4

## Abstract

Rheumatoid arthritis is a chronic inflammatory disease driven by abnormal protein modifications. These include citrullination of arginine residues by the calcium-activated enzyme peptidylarginine deiminase 4 (PAD4). However, calcium in body fluids may not fully activate PAD4, suggesting the potential involvement of other activators. In this study, we investigated the ability of glycosaminoglycans (a class of negatively charged polysaccharides) to modulate PAD4 activity. We found that model glycosaminoglycans bind to the enzyme with a nanomolar affinity, increase its calcium sensitivity, and require enzyme dimerization for activation. These effects depend on the size and negative charge of the glycosaminoglycan, and its various natural forms activate PAD4. Thus, our findings elucidate a mechanism by which common physiological compounds modulate PAD4 activity, potentially contributing to disease etiology.

## Significance

Rheumatoid arthritis is an autoimmune disease characterized by the presence of antibodies against citrullinated epitopes. The source of such peptides and trigger leading to the disease onset are still under debate. In this paper, we show that glycosaminoglycans (negatively charged polysaccharides, like heparin, heparan sulfate, and chondroitin sulfates) can increase the activity of peptidylarginine deiminase 4 (PAD4), enzyme modifying arginine residues in proteins to citrulline. Activation of this enzyme is dependent on the calcium ions, however their concentrations are not sufficient in the human body. Presence of glycosaminoglycans, especially in the joints, could increase the production of citrullinated epitopes leading to and sustaining the autoimmune response.

Posttranslational modifications of proteins play critical roles in both health and disease. Citrullination, the loss of a positive charge on the arginine side chain to form citrulline, is catalyzed by peptidylarginine deiminase 4 (PAD4) of the PAD enzyme family (1). PAD4 is implicated in rheumatoid arthritis (RA), where citrullinated epitopes serve as important diagnostic and pathological factors (2–4). In neutrophil-like cells, PAD4 citrullinates regulatory proteins and RA autoantigens (5), and is crucial for neutrophils extracellular trap (NET) formation (NETosis - process where NETs are released to capture pathogens) as it is necessary for chromatin decondensation via histone citrullination (6–9). This is facilitated by the nuclear localization signal (NLS) in PAD4, unique among human PAD enzymes (10). The active form of PAD4 is a homodimer, and dimerization greatly affects enzyme activity because monomeric mutants are less active (11–13). Enzyme activity also depends on calcium ion concentration. A PAD4 monomer has five binding sites for Ca^2+^, and its saturation results in conformational changes, leading to maturation of the active site (14–16). Half-maximal activation of PAD4 is achieved at calcium concentrations of 300 to 600 µM in biochemical assays (15, 16); physiological free calcium levels reach approximately 1 mM in blood, but are much lower in resting cells (17, 18). Activity of PAD in synovial fluid could be further increased by addition of calcium, suggesting that the enzyme may not be saturated by natural calcium levels and serum of some RA patients could specifically increase PAD4 activity (19, 20). Injection of susceptible mice with PAD4 triggers autoantibodies against citrullinated proteins, and PAD4/2 activity was found in the synovial fluid of patients with RA, which suggests extracellular action (20, 21). Blood glycosaminoglycan (GAG; sulfated, negatively charged polysaccharides) levels correlate with RA severity in humans, and GAG injections induce RA-like symptoms in mouse models, suggesting that GAGs contribute to RA onset (22, 23).

Considering the role of GAGs in RA initiation and progression, and the incomplete activation of PAD4 by body fluids, we hypothesized that GAGs, as exemplified by heparin, directly modulate PAD4 activity, allowing excessive activation at physiologically available Ca^2+^ concentrations.

## Results

### Heparin Binds and Activates PAD4.

**Fig. 1.**
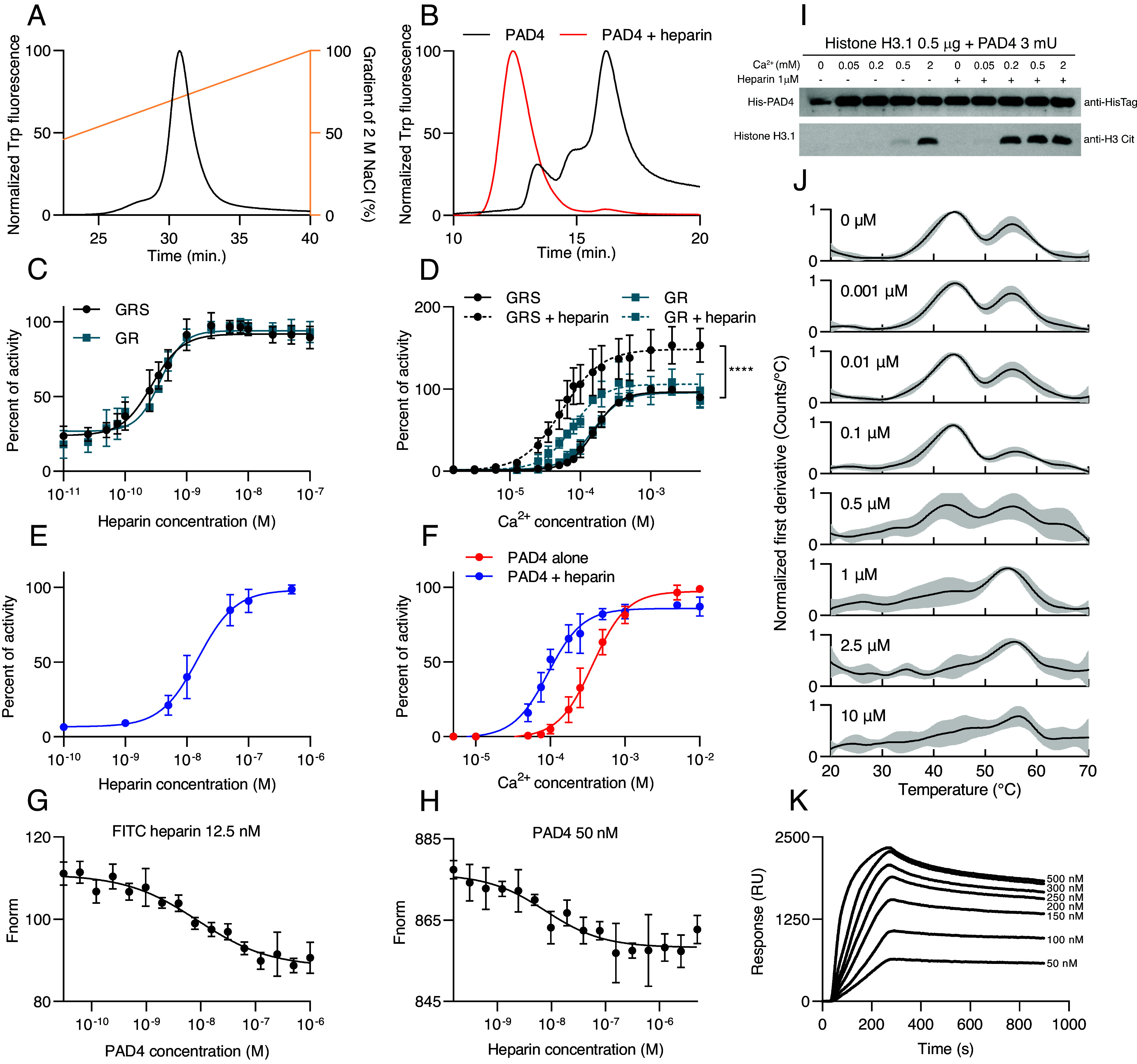
Binding and activation of peptidylarginine deiminase 4 (PAD4) by heparin. (*A*) Elution profile of PAD4 from heparin column in a salt gradient monitored by intrinsic tryptophan fluorescence. (*B*) Size exclusion chromatography of PAD4 on a Superdex 200 column. PAD4 elutes in three forms: monomeric, dimeric, and tetrameric. After the addition of 5 µM heparin, PAD4 elutes in a high molecular weight complex. (*C* and *D*) Activity of PAD4 on substrates Dansyl-GR or Dansyl-GRS, measured using HPLC; data are presented as mean ± SD. (*C*) PAD4 activity at subactivatory Ca^2+^ concentration (0.1 mM) in the presence of increasing heparin concentrations, normalized to the maximal activity in the presence of heparin. Apparent K_D_ values = 0.27 ± 0.02 nM and 0.37 ± 0.03 nM for GRS and GR substrates, respectively. (*D*) Activation of PAD4 by Ca^2+^ with/without addition of 0.025 µM heparin normalized to PAD4 activity in the presence of 2 mM Ca^2+^. Ca_0.5_ values for each substrate and heparin conditions: GRS, 156.8 ± 4 µM; GRS + heparin, 55.1 ± 4.6 µM; GR, 148.2 ± 6.4 µM; GR + heparin, 77.4 ± 5.3 µM. Statistical significance of difference in activity at 5 mM Ca^2+^ with and without heparin for GRS peptide was tested with the unpaired *t* test, ****P < 0.0001, n = 6. (*E* and *F*) Activity of PAD4 on BAEE substrate and colorimetric detection of citrulline; data are presented as mean ± SD. (*E*) Activation of PAD4 by heparin in 0.1 mM Ca^2+^ normalized to maximal activity in the presence of heparin, apparent K_D_ = 17.1 ± 2.9 nM. (*F*) Activation of PAD4 by Ca^2+^ with and without 1 µM heparin, normalized to maximal activity in the presence of calcium. Ca_0.5_ values: without heparin, 370 ± 26 µM; with heparin, 101 ± 19 µM. (*G* and *H*) Microscale thermophoresis binding curves of PAD4 and heparin; data are presented as mean ± SD. (*G*) Fluorescent FITC-heparin was at a constant concentration of 12.5 nM and PAD4 was titrated, in (*H*) PAD4 was kept at 50 nM and heparin was titrated, K_D_ = 8.4 ± 3.0 nM and 9.4 ± 5.3 nM for (*G* and *H*), respectively. (*I*) Western blot assay of PAD4 citrullination of human histone H3.1 at increasing Ca^2+^ concentrations with or without 1 µM heparin. Blot against HisTag of PAD4 is presented as control. (*J*) Thermal unfolding curves of PAD4 with increasing heparin concentrations as indicated, measured by nanoDSF. Data are shown as mean ± SD. (K) Sensograms from a representative SPR interaction assay of increasing PAD4 concentrations with heparin immobilized on the chip surface. Binding was analyzed using a one-site model, yielding K_D_ = 4.9 ± 1.1 nM. Values after ± indicate SEM.

Heparin chromatographic resins are used as ion exchange or specific affinity media for GAG-interacting proteins. We therefore tested the binding of recombinant human PAD4 to an analytical heparin column (Fig. 1*A*). The protein eluted at approximately 1.4 M salt concentration, higher than expected for ion-exchange interactions (24), suggesting specific, high-affinity binding. We verified this observation using size exclusion chromatography of PAD4 alone and in the presence of full-length heparin. Several peaks in the PAD4-alone elution profile were observed, which corresponded with monomers (containing the majority of proteins), dimers, and a larger form (possibly a tetramer). However, after the addition of heparin, most of the PAD4 was eluted as a high molecular weight complex, larger than any form of PAD4-alone, whereas the monomeric form was detected in small quantities (Fig. 1*B*). The estimated molecular weight of this heparin-induced complex (approximately 400 kDa) was consistent with that of a GAG-bound, tightly packed hexamer of PAD4. To investigate the potential activity of this PAD4 complex, we measured its enzymatic activity using HPLC with fluorescently labeled di- or tripeptide (GR and GRS, respectively) substrates (25, 26) at a subactivatory Ca^2+^ concentration (0.1 mM). Heparin activated PAD4 with an apparent K_D_ < 1 nM (Fig. 1*C*) for both substrates. We verified the potential activation mechanism by testing PAD4 calcium dependence in the presence of an activating heparin concentration (0.025 µM). The half-activatory calcium concentration Ca_0.5_ decreased for both peptide substrates, from 156.8 and 148.2 µM to 77.4 and 55.1 µM without and with heparin, respectively (Fig. 1*D*). Additionally, heparin increased maximum activity even under fully saturating calcium concentrations on GRS peptide. We further validated these results using a colorimetric PAD4 activity assay with the small-molecule substrate BAEE. The assay showed an apparent K_D_ of 17.1 nM and Ca_0.5_ of 101 µM in the presence of heparin (1 µM), compared to 370 µM in its absence (Fig. 1 *E* and *F*).

We directly measured the PAD4–heparin interaction using microscale thermophoresis in two alternate settings: 1. the signal from FITC-labeled heparin interacting with unlabeled PAD4 and 2. the intrinsic tryptophan fluorescence of PAD4 complexing with unlabeled heparin. A K_D_ of approximately 9 nM was obtained in both cases (Fig. 1 *G* and *H*). These values are consistent with the surface plasmon resonance (SPR) results showing a K_D_ of 4.9 nM (Fig. 1*K*).

In a histone H3 citrullination assay, addition of heparin allowed efficient citrullination of substrate at 200 µM Ca^2+^, whereas 2 mM Ca^2+^ was required to reach similar levels of modification in the absence of heparin (Fig. 1*I*). Next, we tested the impact of heparin on PAD4 thermal stability with nanoDSF. Heparin stabilized PAD4, shifting its melting curve from two distinct unfolding events (44 and 55 °C) for PAD4 alone to a single unfolding event (55 °C) at 1 µM heparin (Fig. 1*J*). This observation suggests that PAD4 undergoes two distinct unfolding events when heparin is absent: an initial disruption of its multimeric structure at 44 °C, followed by complete protein unfolding at 55 °C. In the presence of heparin, these multimeric forms are stabilized, resulting in a single unfolding event at 55 °C. Heparin also provided additional stabilization when PAD4 was titrated with calcium (*SI Appendix*, Fig. S1). Both heparin and calcium protected PAD4 from proteolytic degradation by trypsin, RgpB, Kgp, and V8 protease, albeit the protection pattern differed, confirming the stabilizing effect of both binders on PAD4 (*SI Appendix*, Fig. S2).

### Effect of Heparin Depends on Chain Length and Charge.

**Fig. 2.**
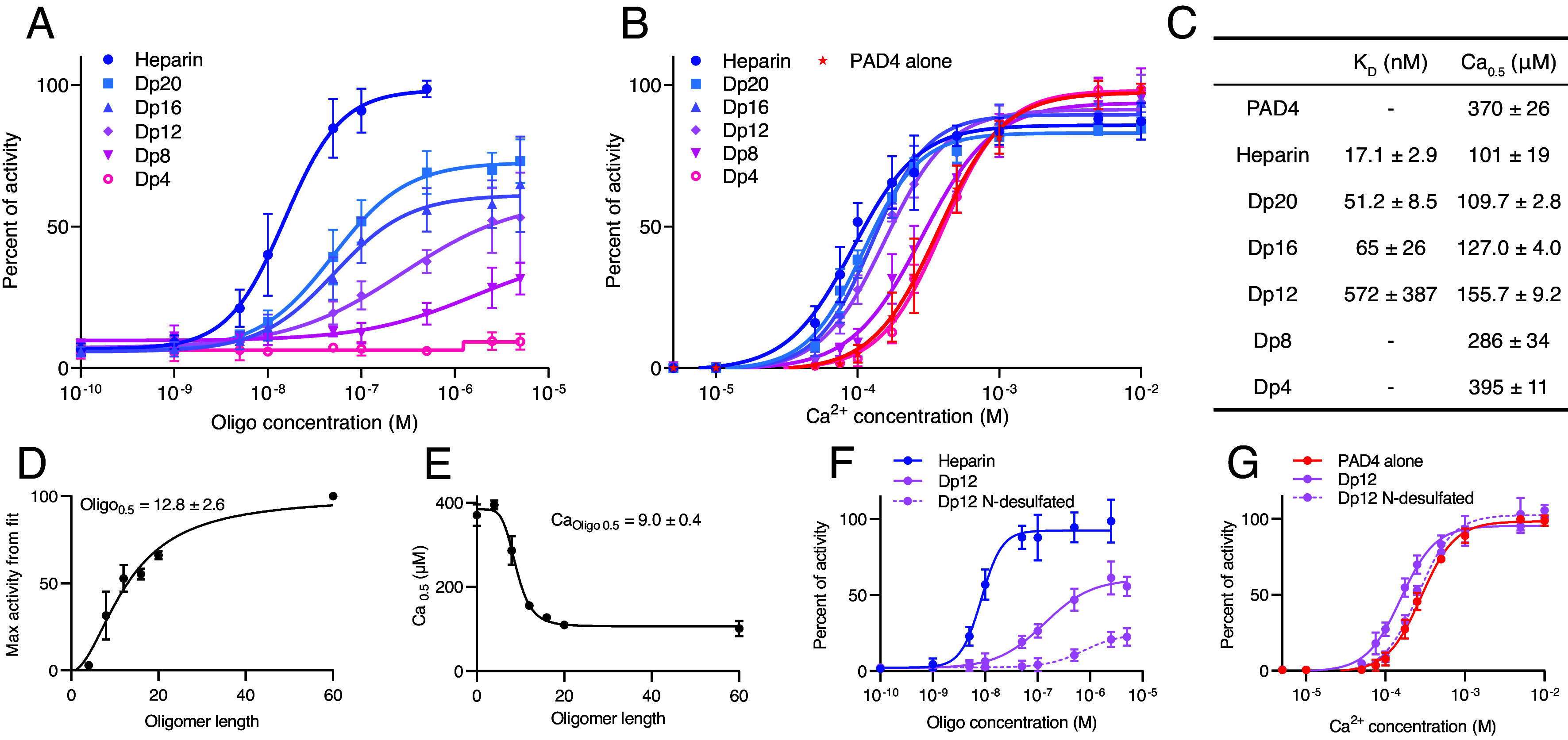
Effect of chain length and charge of heparin oligomers on PAD4 activation. (*A* and *B*) Activity of PAD4 measured on BAEE substrate with colorimetric detection of produced citrulline, data are presented as mean ± SD. (*A*) Activity of PAD4 in the presence of 0.1 mM Ca^2+^ and heparin oligomers with decreasing length (4 to 20 monomers for Dp4–Dp20, respectively). The apparent K_D_ values are summarized in (*C*). (*B*) Activation of PAD4 by calcium in the presence of 1 µM heparin oligomers of different lengths. Ca_0.5_ values are shown in (*C*). (*C*) Summary of apparent K_D_ and Ca_0.5_ values for heparin oligomers from (*A* and *B*). (*D* and *E*) Maximal activity of PAD4 from fits in (*A*) and Ca_0.5_ from (*B*) plotted against oligomer length. Hill equation was fitted to determine the length of the oligomer at which half of the effect is achieved. Error bars represent SEM of fitted values. (*F* and *G*) Activity of PAD4 in the presence of heparin oligomer with 12 subunits (Dp12), its N-desulfated and reN-acetylated (Dp12 N-desulfated) form, and heparin (for comparison), measured using a BAEE colorimetric assay; data are presented as mean ± SD. (*F*) Activity in 0.1 mM Ca^2+^ and increasing concentrations of GAGs, apparent K_D_ values: heparin, 8.6 ± 0.4; Dp12, 169 ± 56; and Dp12 N-desulfated, 3,041 ± 1,709 nM. (*G*) Activation of PAD4 with Ca^2+^ in the presence of 1 µM of GAGs, Ca_0.5_ values: no GAG, 281.2 ± 7.4; Dp12, 157 ± 12; Dp12 N-desulfated, 258.6 ± 8.0 µM. Values after ± indicate SEM.

After determining the activatory effect of heparin (which is, on average, approximately 60 monomers in length) on PAD4, we investigated the properties of the GAG polymer that contribute to this phenomenon. We employed short heparin chain fragments of defined lengths (Dp4–Dp20, corresponding to 4 to 20 monomers) to verify the size of the molecule required to effectively activate PAD4. In a colorimetric assay with the BAEE substrate, we determined that the apparent affinity of heparin oligomers to PAD4 decreased with shortening of the heparin chain, in concordance with the maximum level of activation (Fig. 2 *A* and *C*). Similarly, the increase in calcium affinity was less prominent in the presence of smaller heparin fragments (Ca_0.5_ = 101 µM with full heparin and 395 µM with Dp4, compared to 370 µM without heparin) (Fig. 2 *B* and *C*). By plotting the maximum PAD4 activation and Ca_0.5_ versus (vs) oligomer length, we determined that approximately 12 sugar subunits in the GAG chain are necessary to achieve half of the effects of native heparin (Fig. 2 *D* and *E*). Furthermore, we tested the N-desulfated variant of a 12-subunit heparin oligomer (Dp12_ND_), which was a much weaker PAD4 activator compared to the unmodified oligomer (Dp12) with full negative charge (apparent K_D_ = 3,040 nM for Dp12_ND_ vs 168 nM for Dp12; Ca_0.5_ = 258.6 µM for Dp12_ND_ vs 156 µM for Dp12) (Fig. 2 *F* and *G*).

### Structures of PAD4 with Heparin Oligomers.

**Fig. 3.**
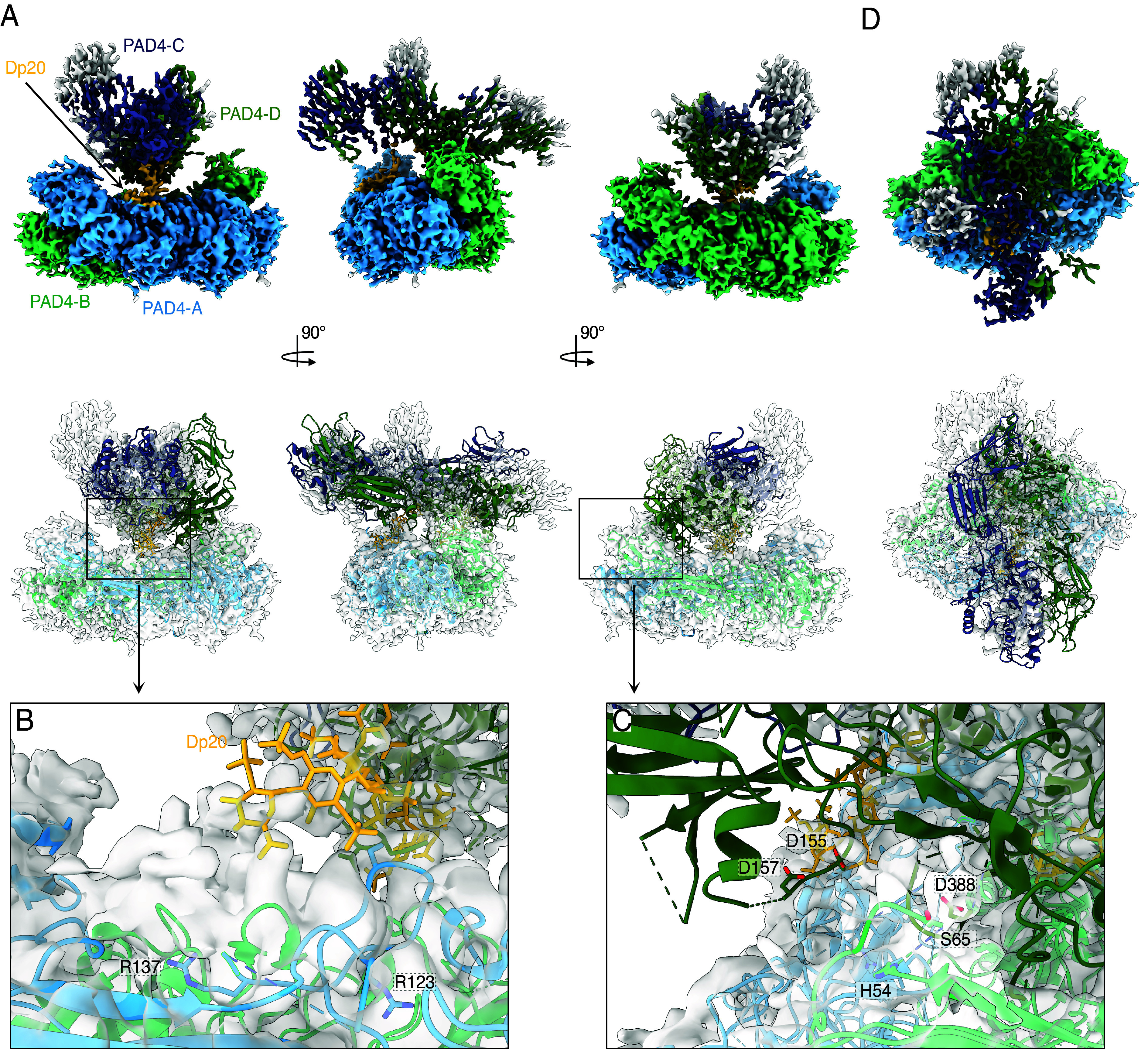
Cryoelectron microscopy structures of PAD4 in complex with the Dp20 heparin oligomer in the presence of 0.1 mM CaCl_2_. Quality of the map was insufficient to confidently model the protein, therefore our model of PAD4 with Dp20 in the absence of calcium was fitted in the map (PDB: 9HUI). (*A*) CryoEM map and model in cartoon representation with map overlaid, colored by chain (green and blue: chains *A* and *B* from the *Bottom* dimer; *C* and *D* from the *Top* dimer). The excessive density, assigned to flexible GAG chains is colored yellow, with a tentative model shown as sticks for visualization. (*B* and *C*) Close-up panels showing (*B*) residues at the ends of flexible loop not resolved in the model proposed to interact with the Dp20 oligomer; (*C*) interaction between the nuclear localization signal of the *Bottom* dimer and calcium binding region of the *Top* dimer, ends of loop containing NLS (H54-S65) are marked, the loop itself is not resolved in the model. (*D*) *Top* view of structures from (*A*) showing the 90° double-propeller, cross-like structure.

Heparin preparations from natural sources have a wide distribution of molecular sizes owing to the heterogeneity of this molecule. Therefore, we opted to use short oligomers of defined lengths for structural studies. Cryoelectron microscopy was used to obtain the structure of PAD4 with heparin oligomers (Table 1). PAD4 activation was observed at 0.1 mM CaCl_2_ in our assays; therefore, we aimed to determine the PAD4–GAG complex structure under these conditions using Dp20 (*SI Appendix*, Fig. S3, S4). The obtained map showed two dimers of PAD4 connected by two Dp20 chains bound proximal to the region spanning a flexible, unresolved loop, including R123 to R137, and rich in positively charged residues (123-RTGKVKPTRAVKDQR-137). The two dimers were stacked face-to-back and angled close to 90° in the x–y plane, forming a cross-like double-propeller structure (Fig. 3). In the x–z plane, the top dimer lies nearly flat on the bottom dimer. This organization allows interaction between the NLS region of the lower dimer (K59, K60, and K61) and the region near the Ca3-5 calcium-binding sites of the top dimer. However, resolution constraints hindered the precise definition of the effects of direct dimer–dimer binding. The calcium-binding sites remained unresolved, even in our 10 mM CaCl_2_ structure of PAD4 (*SI Appendix*, Figs. S5–S7). We also determined the structure of PAD4 in the presence of Dp20 and in the absence of calcium (*SI Appendix*, Figs. S8–S10). In this case, different structures were formed, consisting of a single PAD4 dimer with GAG oligomers spanning across the dimer to bridge two regions proximal to the N-terminal of the protein (“handbag” like structure). Importantly, these interaction anchors between the GAG and PAD4 were located near the contact surface between the monomers, encompassing the positively charged NLS. Additionally, as inferred from the density map, a GAG chain in the correct conformation can make contact with a loop containing R123.

**Table 1.** Cryo-EM data collection, refinement, and validation statistics

Map	PAD4 + Dp20 in 0.1 mM Ca^2+^ EMD-52412	PAD4 in 10 mM Ca^2+^ EMD-52413 PDB: 9HUH	PAD4 + Dp20 EMD-52414 PDB: 9HUI	PAD4 + Dp12 EMD-52415 PDB: 9HUJ
**Data collection and processing**				
Microscope	TFS KRIOS	TFS KRIOS	TFS KRIOS	TFS KRIOS
Voltage (kV)	300	300	300	300
Detector	GATAN K3 BIOQUANTUM (6 × 4 k)	GATAN K3 BIOQUANTUM (6 × 4 k)	GATAN K3 BIOQUANTUM (6 × 4 k)	GATAN K3 BIOQUANTUM 6 × 4 k)
Magnification	105,000	105,000	105,000	105,000
Pixel size (Å/pixel)	0.8456	0.8456	0.8456	0.8456
Exposure (e^−^/ (Å^2^)	40	40	40	40
Defocus (µm)	0.7 to 2.5	0.7 to 2.5	0.7 to 2.5	0.7 to 2.5
Particles used	91750	93239	209466	235606
Map symmetry	–	C2	C1	C1
Map resolution (Å)	3.2	2.96	3.73	3.57
**Structure validation**				
Protein residues	–	1144	1127	2225
RMSZ bonds	–	0.50	1.02	0.33
RMSZ angles	–	0.66	0.76	0.57
Ramachandran plot	–			
Favored (%)	–	93	94	95
Allowed (%)	–	6	5	5
Outliers (%)	–	0.5	0.3	0.0
Rotamer outliers (%)	–	4.2	3.9	2.4
Clashscore	–	14	25	12

To explain the lower activatory potential of shorter oligomers, we determined the structure of PAD4 with Dp12 in the absence of calcium (*SI Appendix*, Figs. S11–S13). This structure also contained two face-to-back dimers of PAD4, similar to the Dp20-bound structure, but they were connected by a single Dp12 and angled differently (approximately 73° in the x–y plane and approximately 38° in the z–x plane). The Dp12 oligomer was again bound proximal to the region spanning a loop including R123 to R137 of the bottom dimer. In addition, one more Dp12 molecule could reside in loop R123–R137 on the distal side of the top dimer, possibly allowing for the formation of longer PAD4 dimer chains. These structures were observed in the micrographs as a separate 2D class (*SI Appendix*, Fig. S14). The data demonstrate that heparin oligomers bind PAD4 in two different modes, depending on the GAG molecule size and the presence of calcium ions. Both involve regions rich in positively charged residues at two distal locations, indicating that heparin binding is mediated by a dispersed interaction interface rather than by a defined allosteric pocket on the enzyme surface.

### Mechanism of PAD4 Activation by Heparin.

Based on the structural data, we identified the PAD4 sites potentially responsible for the binding and/or activation of the enzyme by heparin. We prepared mutants of positively charged residues in the suspected regions:

1. PAD4^123/126/128^: R123S K126S K128S.
2. PAD4^126/128/131/134/137^: K126S K128S R131S K134S R137S.
3. PAD4^59/60/61/81/91^: K59S K60S K61S K81S K91S.

**Fig. 4.**
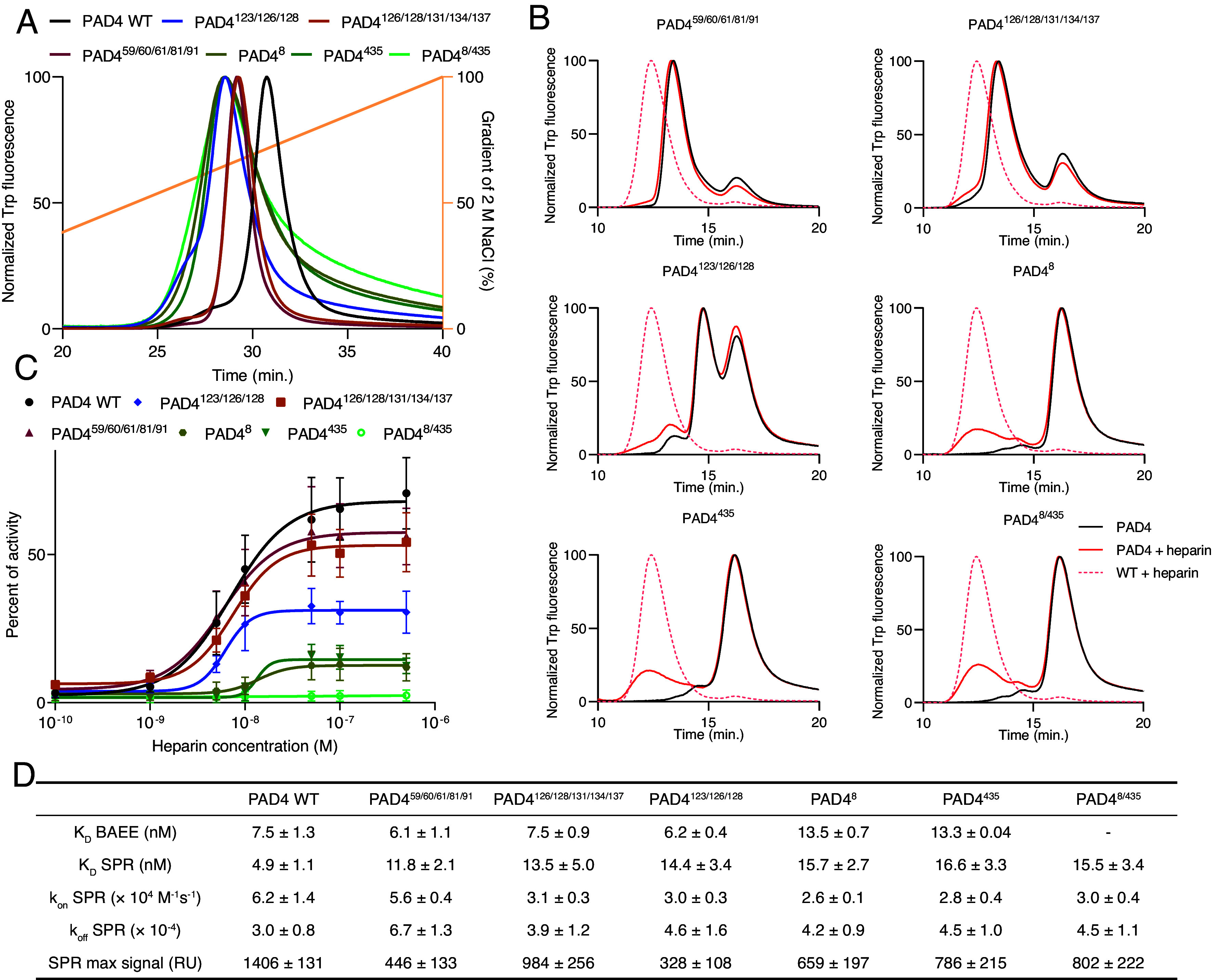
Characterization of heparin binding to PAD4 mutants. (*A*) Elution profiles of PAD4 WT and mutants from a Poros heparin column in a salt gradient monitored by intrinsic tryptophan fluorescence. (*B*) Size exclusion chromatography of PAD4 mutants with and without 5 µM heparin on a Superdex 200 column monitored by tryptophan fluorescence. PAD4 WT with heparin is shown for reference. (*C*) Activation of PAD4 and its mutants by heparin at 0.1 mM Ca^2+^ using BAEE substrate. Data are presented as percent of activity of each mutant in 5 mM Ca^2+^; mean ± SD. Hill equation was fitted to calculate apparent K_D_ for each mutant shown in (*D*), together with K_D_, k_on_, k_off_, and maximum signal from SPR assay of interaction with immobilized heparin. The mutation regions include: PAD4^123/126/128^, R123S K126S K128S; PAD4^126/128/131/134/137^, K126S K128S R131S K134S R137S; PAD4^59/60/61/81/91^, K59S K60S K61S K81S K91S and dimerization regions PAD4^8^: R8E; PAD4^435^: Y435A, and PAD4^8/435^: R8E Y435A. Values after ± indicate SEM.

We tested their binding to an analytical heparin column, and found that all mutants eluted earlier in the gradient compared to wild-type (WT) PAD4, suggesting weaker binding (Fig. 4*A*). Accordingly, when analyzed by size exclusion chromatography, the mutants showed only subtle changes in the elution profiles after the addition of heparin, with limited formation of larger PAD4 oligomers in the PAD4^123/126/128^ mutant. The slight mass increase after the addition of heparin in PAD4^126/128/131/134/137^ and PAD4^59/60/61/81/91^ suggests the binding of heparin, but without the formation of complexes larger than tetramers. Interestingly, these mutants eluted predominantly as tetramers and minute amounts of monomers, even in the absence of heparin; no dimers were observed (Fig. 4*B*).

The PAD4^123/126/128^ mutant showed weaker activation by heparin at low calcium concentrations, as illustrated by the low maximal activity in the presence of heparin. However, the apparent K_D_ values were similar to those of the WT (PAD4^123/126/128^, 6.2 nM; WT, 7.5 nM) (Fig. 4 *C* and *D*). Meanwhile, the PAD4^126/128/131/134/137^ and PAD4^59/60/61/81/91^ mutants were activated marginally weaker than WT and retained an apparent K_D_ close to that of the WT (PAD4^126/128/131/134/137^, 7.5 nM; PAD4^59/60/61/81/91^, 6.1 nM). The K_D_ values were confirmed by SPR analysis, showing similar heparin affinity for the mutants as that for WT but a reduced SPR response (Fig. 4*D*). Together, these results indicate that the mutation at PAD4^123/126/128^ has a measurable but limited effect on PAD4 activation, whereas those at PAD4^126/128/131/134/137^ and PAD4^59/60/61/81/91^ do not. Notably, the PAD4^126/128/131/134/137^ mutant includes the same mutations as the PAD4^123/126/128^ mutant, except for R123S, suggesting that this residue might be directly involved in activation.

PAD4 activity is dependent on protein dimerization (11, 12). Therefore, we tested the activation of monomeric PAD4 (11–13) by heparin using dimerization-impaired PAD4 mutants:

4. PAD4^8^: R8E.
5. PAD4^435^: Y435A.
6. PAD4^8/435^: R8E Y435A (double mutant).

All three dimerization mutants were eluted earlier than the WT from the heparin column (Fig. 4*A*). Size exclusion analysis showed that the three mutants were almost exclusively present as monomers, with a minor dimeric fraction (also confirmed by dynamic light scattering analysis of the molecular size; *SI Appendix*, Fig. S15). Addition of heparin resulted in the detection of only a small amount of larger heparin-bound complexes (Fig. 4*B*). Furthermore, in the activation assay, the mutants PAD4^8^ and PAD4^435^ showed weak activation, while the double PAD4^8/435^ mutant was not activated at all, indicating that heparin could not overcome the dimerization requirement. The apparent K_D_ values remained in the low nanomolar range (13 nM for mutants PAD4^8^ and PAD4^435^ vs 7.5 nM for the WT). All three mutants were able to bind to the heparin-coated SPR chip, with K_D_ values slightly higher than those of the WT (Fig. 4*D*). Compared to the WT, the SPR signal response in the mutants was reduced, suggesting potential on-chip binding of only the residual dimer fraction. Importantly, all prepared mutants were expressed and isolated with comparable yields and retained similar specific activity as WT, with the predicted exception of dimerization mutants, which were less active. Also the molecular size in solution was consistent with the expected values (*SI Appendix*, Fig. S15*C*).

### Biological Properties of PAD4 Activation with GAG.

**Fig. 5.**
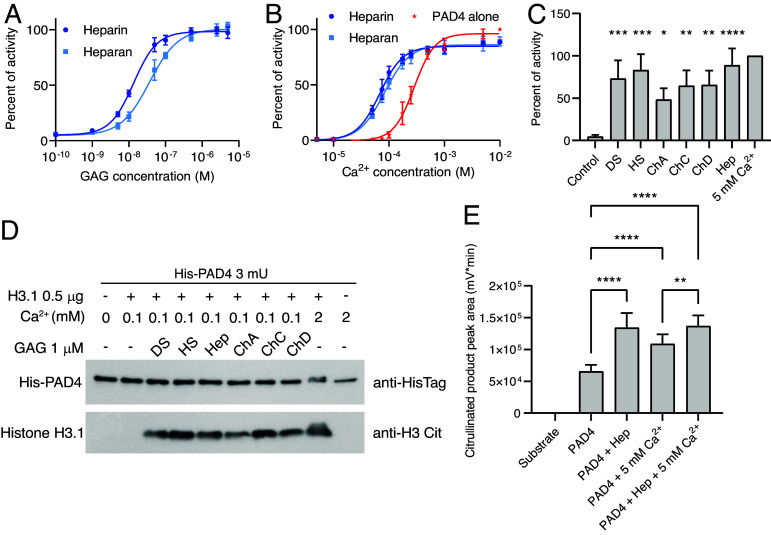
Activation of PAD4 by other natural glycosaminoglycans. (*A* and *B*) Activity of PAD4 in colorimetric assay on BAEE substrate; data are presented as mean ± SD. (*A*) Activation of PAD4 by heparan sulfate in 0.1 mM Ca^2+^ normalized to maximal activity in the presence of heparin. Apparent K_D_: heparin, 13.6 ± 0.9; heparan, 37.9 ± 4.5 nM. (*B*) Activation of PAD4 by Ca^2+^ with and without 1 µM of activator, normalized to maximal activity in the presence of calcium. Ca_0.5_ values: no activator, 285.6 ± 7.0; heparin, 69.2 ± 3.3; heparan, 83.7 ± 4.6 µM. (*C* and *D*) Activation of PAD4 in 0.1 mM Ca^2+^ after the addition of 1 µM of various GAGs. (*C*) Colorimetric PAD4 activity assay with BAEE substrate. Data are presented as percent of activity in 5 mM Ca^2+^; mean ± SD. Statistical significance compared to the control was evaluated using one-way ANOVA with Dunnett multiple comparison correction; *P < 0.05, **P < 0.01, ***P < 0.001, ****P < 0.0001, n = 3. (*D*) Western blot assay of PAD4 citrullination of human histone H3.1. Blot against HisTag of PAD4 is presented as the control; results from representative experiment are shown. DS, dermatan sulfate; HS, heparan sulfate; ChA, chondroitin A; ChC, chondroitin C; ChD, chondroitin D; Hep, heparin. (*E*) Activity of PAD4 on Dansyl-GR substrate measured using HPLC; mean ± SD. PAD4 was added to 90% human serum supplemented with substrate and 1 µM heparin or 5 mM Ca^2+^. Statistical significance was tested using one-way ANOVA with Tukey multiple comparison correction **P < 0.01, ****P < 0.0001, n = 8. Values after ± indicate SEM.

We investigated whether natural GAGs, other than heparin, affect PAD4 activation. We found that heparan sulfate, a GAG present ubiquitously on the surface of cells (27), also binds and activates PAD4 with nanomolar affinity (apparent K_D_ = 37.9 nM) and lowers its calcium activation threshold (Ca_0.5_ = 83.7 µM with heparan sulfate vs 285 µM without) (Fig. 5 *A* and *B*). Furthermore, at 1 µM concentration, dermatan sulfate, heparan sulfate, and chondroitin sulfates A, C, D, all activated PAD4 with varying potency in the presence of 0.1 mM Ca^2+^ (Fig. 5*C*). In fact, dermatan and heparan sulfates activated PAD4 at levels similar to those of heparin, whereas chondroitin sulfate induced lower activity. At 1 µM, all tested compounds stimulated histone H3 citrullination at low Ca^2+^ concentrations, similar to heparin (Fig. 5*D*). We aimed to further confirm the activation of PAD4 by heparin in the human serum. We observed that in 90% human serum the PAD4 activity increased after addition of GAG even in the presence of 5 mM Ca^2+^ and that the potential of heparin to hyperactivate PAD4 was retained (Fig. 5*E*).

## Discussion

Based on reports that GAGs can cause RA-like symptoms, and that body fluids may not fully activate PAD4, we hypothesized that these events may be linked to PAD4 activation (19, 22, 23). Indeed, we showed that heparin activates PAD4 with low nanomolar affinity. The strength of this interaction appears to be the result of a very slow complex dissociation, as demonstrated by SPR analysis. High salt concentrations (1.4 M) were required to break the complex. Activation was achieved by increasing the affinity of PAD4 for calcium, allowing full activation even at 0.1 mM Ca^2+^. Such levels can be found in physiological fluids, where nanomolar concentrations of heparin can trigger PAD4 activation, forming a stable, active complex. What’s more, heparin increased maximum activity of PAD4, beyond what was achieved by calcium saturation alone, when tested on peptide substrates. This excess activation was observed in the human serum, highlighting that heparin can hyperactivate PAD4 in the extracellular environment, where the two molecules could meet.

The distinctive features of heparin include its polymeric structure and strong negative charge, both of which were essential for the activation of PAD4 in our study. Short or undersulfated fragments had a weaker effect on the enzyme, similar to other GAGs interacting with proteins (28). Size-exclusion chromatography revealed that full-length heparin induces the formation of high-molecular-weight, high-hydrodynamic-radius complexes, consistent with a heparin-bound PAD4 hexamer. Mutagenesis studies of suspected PAD4 binding sites were inconclusive. The mutant of the first putative binding site PAD4^123/126/128^ was activated to a limited extent by heparin treatment. Mutants PAD4^126/128/131/134/137^ and PAD4^59/60/61/81/91^ were activated similarly to the WT, but only formed tetramers, and did not shift to hexamers after the addition of heparin. The increased proportion of tetramers in these mutants, even in the absence of heparin, may be explained by the disruption of the positive charge on the protein surface, which normally limits the interaction between the two dimers. Importantly, these mutants were still activated by heparin at a level similar to that of WT. This observation is consistent with the binding of heparin on the dispersed positively charged surfaces of PAD4. The removal of individual positively charged regions had a limited effect on the interaction with heparin. A comparison of the PAD4^123/126/128^ and PAD4^126/128/131/134/137^ mutants suggested that R123 may be an important residue for activation upon heparin binding. Indeed, R123 was previously speculated to regulate the stability and organization of the calcium sites Ca3-4 (29).

PAD4 mutants with impaired dimerization exhibit a more prominent reduction in heparin-induced enzyme activation. Therefore, we propose that heparin binds to already dimerized PAD4, resulting in the formation of stable complexes and the lowering of the calcium activation threshold through allosteric regulation. The observed change in the dimer–dimer organization between Dp12 and Dp20 at 0.1 mM Ca^2+^ consists of the introduction of an additional GAG anchor point for a longer Dp20 oligomer, which introduces torsion sufficient to pull the dimers together and form the 90° double-propeller, cross-like structure. This, in turn, allows for the interaction between the NLS, located in the N-terminal domain of the lower dimer, and the calcium sites Ca3-5 located on the side of the top dimer. This explains why longer oligomers are more effective activators, as they allow a sufficient distance between the two dimers to utilize the two attachment sites. Simultaneously, R123- and NLS-mediated stabilization of the Ca3-5 sites may explain the increased calcium affinity of the formed complex. Similar observations were made for neutrophil elastase binding of heparin, which also involved diffuse binding surfaces and bridging protein dimers. The same study also highlights the challenges of structural analysis of heparin–protein complexes arising from heterogeneity of heparin chains (30). This mode of interaction is significantly different than the well-defined and highly specific interaction between heparin and some other protein partners, e.g. thrombin–heparin–antithrombin III complex (31). Additionally, binding of heparin could have stabilizing and protective effect on PAD4, as the thermal stability and proteolysis resistance of the complex was increased compared to free protein. Formation of large complexes of GAG-bound PAD4 can also result in the accumulation of active PAD4 at the site of the inflammatory reaction that triggered PAD4 release from the cells.

Previous studies have revealed that some patients with RA possess cross-reactive anti-PAD3/PAD4 antibodies, which activate enzymes by increasing calcium affinity and promote disease progression (32–34). This mode of PAD4 activation was also recently investigated by generating synthetic activatory cyclic peptides that bind to calcium sites Ca3-5 (35). In this study, we identified GAGs as physiological allosteric coactivators of PAD4. While anti-PAD3/4 activatory antibodies are present in only a minor fraction of patients with RA, PAD4 activation by GAGs released locally from the cartilage during an inflammatory response could contribute to RA development in a wider population of patients. Heparin was also observed to induce NETosis and some reports claim that it can enter the cells (36, 37), hence activation of PAD4 intracellularly could be hypothesized, however this requires further research.

Study presented here shows activation of PAD4 by GAGs under physiological conditions and explains this by increased calcium sensitivity resulting from the formation of GAG–PAD4 complexes. With the limited resolution of the present structural data precluding us from the precise determination of GAG-binding details, we aim to demonstrate the overall architecture of heparin-mediated supramolecular complex. Due to the limited resolution of our structures and heterogenic nature of GAG oligomers we were not able to provide the full structural explanation and directly demonstrate the improved organization of the calcium binding sites or active center of PAD4. Maps in the region of the “upper” PAD4 dimer cannot be interpreted in detail (*SI Appendix*, Fig. S4, S12), allowing us only to place the second dimer in respect to the first one, using the regions, where the quality is reasonably good. The map quality is not sufficient to provide a reasonable model reconstruction. Therefore, we do not interpret the “upper” model and do not infer any details regarding GAG interaction on the “back” side of the “upper” dimer. The question whether GAG binding improves calcium affinity of Ca1-Ca5 sites, or may it replace the necessity of calcium at some of these sites remains open and will be subjected to our future research.

The biological importance of our findings is highlighted by the observation that PAD4 activation is not unique to heparin. Other GAGs have similar properties, including the widely present heparan sulfate and chondroitin sulfate, which may be relevant to RA. We hypothesize that in RA pathology, an initial inflammatory imbalance leads to the release of GAGs from the cartilage and PAD4 from neutrophils, which in turn provoke the hyperactivation of PAD4 even at saturating calcium levels, exacerbating the production of citrullinated peptides in the synovial fluid. Our data hint at this possibility, as heparin further activated PAD4 even in human serum supplemented with 5 mM calcium, a concentration much higher than physiological. This increased activation of PAD4, released from neutrophils, by extracellular GAGs might be an early step leading to RA development in susceptible patients after the initial inflammatory trigger, as well as a driver of prolonged joint destruction over the course of the disease.

## Materials and Methods

### Recombinant PAD4 Expression and Purification.

Full length human PAD4 sequence (GenBank NM_012387) with N-terminal affinity Histidine tag cloned into pET16b vector was synthesized by GenScript Biotech Corporation. Subsequent mutant variants of PAD4 were also synthesized: 1. PAD4^123/126/128^ (R123S K126S K128S); 2. PAD4^126/128/131/134/137^ (K126S K128S R131S K134S R137S); 3. PAD4^59/60/61/81/91^ (K59S K60S K61S K81S K91S); 4. PAD4^8^ (R8E); 5. PAD4^435^ (Y435A); 6. PAD^48/435^ (R8E Y435A). Plasmids were transformed into *Escherichia coli* BL21(DE3) expression strain using heat shock protocol. Preculture was diluted 50x in 800 ml of fresh LB with 100 µg/ml ampicillin and incubated until OD_600_ reached 0.6. Expression was induced with 0.5 mM IPTG (isopropyl β-D-thiogalactopyranoside, BioShop #IPT001) and carried for 16 h at 26 °C with 180 rpm shaking. Centrifuged cells were sonicated for 3 min on Sonics VCX500 sonicator. Lysate was then centrifuged and clarified supernatant was loaded on a 5 ml HisTrap FF column (Cytiva #17531901) connected to Akta Pure system (Cytiva). Column was then washed and protein was eluted with linear gradient 0 to 100% of 500 mM imidazole. Fractions containing PAD4 were then subjected to buffer exchange. PAD4 was loaded on an HiTrap Heparin HP column (Cytiva #17040601), washed with 10 column volumes of binding buffer and eluted with linear gradient 0 to 100% of 2 M NaCl. Protein containing fractions were pooled and subjected to size exclusion chromatography on HiLoad 16/600 Superdex 200 pg column (Cytiva #28989335) in 50 mM Tris pH 7.5, 300 mM NaCl. Finally, protein was concentrated on Amicon Ultra 15, 10000 NMWL (Millipore #UFC901024). Detailed description of the purification procedure is provided in *SI Appendix*.

### Affinity Chromatography of PAD4 and Its Mutants On Heparin Column.

Binding of PAD4 and its mutants to heparin column was performed on a POROS Heparin 50 μm 2.1 × 30 mm column (Thermo Scientific #4333411) connected to a Shimadzu Nexera 2 chromatography system. PADs at 0.25 µM concentration were applied on column in buffer: 50 mM Tris pH 7.5, 150 mM NaCl and resolved in 7.5 to 100% gradient of phase B (50 mM Tris, pH 7.5, 2 M NaCl) over 30 column volumes. Tryptophan fluorescence of samples was monitored at excitation wavelength of 280 nm and emission wavelength of 350 nm.

### Size Exclusion (SEC) Analysis of PAD4 and Its Mutants in Complexes.

Analytical SEC of PAD4 and its mutants in the presence or absence of 5 µM heparin (Heparin sodium salt from porcine intestinal mucosa, Sigma-Aldrich #H3149) was performed on a Superdex 200 Increase 3.2/300 column (Cytiva, #28990946) connected to a Shimadzu Nexera 2 chromatography system. Each protein (30 µl) was run at 0.15 µM concentration in running buffer consisting of 50 mM Tris pH 7.5, 300 mM NaCl, 0.1 mM CaCl_2_, 0.05% Tween20, 0.25 mM DTT. Tryptophan fluorescence of samples was monitored at excitation and emission wavelength of 280 and 350 nm respectively. Retention time of each peak was compared with protein standards from the HMW Gel Filtration Calibration Kit (Cytiva #28403842).

### RP-HPLC-Based Method for Measurement of PAD4 Activity in the Presence of Different Calcium and Heparin Concentrations.

PAD4 activity in the presence of different CaCl_2_ or heparin concentrations was measured with RP-HPLC-based method. PAD4 (0.25 mU) was incubated with CaCl_2_ concentrations 0 to 5 mM (calcium curve) in the presence or absence of 0.025 µM heparin (Heparin sodium salt from porcine intestinal mucosa, Sigma-Aldrich #H3149); or heparin concentrations 0 to 0.1 µM (heparin curve) in the presence of 0.1 mM CaCl_2_. Fluorescently labeled substrate was used at 100 µM: Dansyl-Gly-Arg or Dansyl-Gly-Arg-Ser (both custom synthesized by PeptideWeb) in reaction buffer (100 mM Tris pH 7.5, 5 mM DTT, 1% DMSO), samples were incubated for 1 h at 37 °C in 50 µl total volume. Reaction was stopped by the addition of 5 µl 1% TFA. PAD4 activity in the presence of 90% normal human serum (Complement Technologies) was measured with RP-HPLC-based method. PAD4 (1 mU) was incubated with 90% NHS, 100 µM Dansyl-Gly-Arg substrate, 5 mM DTT, 2 mM orthophenanthrolin (Sigma-Aldrich) in the presence or absence of 1 µM heparin. Additionally, a set of samples supplemented with additional 5 mM CaCl_2_ was prepared. Samples were incubated for 1 h at 37 °C in 30 µl total volume. Reaction was stopped by the addition of 40 µl 5% TCA and precipitated on ice for 15 min. Additional 35 ul of TFA was added and samples were centrifuged 15 min, 16,000 g at 4 °C. Samples were resolved on an Aeris 2.6 µm Peptide XB-C18 50 × 2.1 mm column (Phenomenex #00B-4505-AN) connected to a Nexera 2 chromatography system (Shimadzu) in gradient of phase A (0.1% TFA in water) and B (80% acetonitrile, 0.08% TFA in water) for Dansyl-GR 5 to 20%, for Dansyl-GRS 5 to 30% of phase B over 26 column volumes. Dansyl-label fluorescence was monitored at excitation wavelength of 333 nm and emission wavelength of 533 nm. Area of the peaks corresponding to the citrullinated products was calculated with LabSolution Software 5.99 SP2 (Shimadzu). Results are shown as a percent of PAD4 activity on each substrate in 2 mM CaCl_2_ without heparin for calcium curve; and as percent of maximal PAD4 activity on each substrate in the presence of heparin for heparin curve.

### PAD4 Activity Assay With BAEE Substrate.

PAD4 activity was measured with spectrophotometric COLDER Assay (38). For general evaluation of PAD4 activity, enzyme was incubated in buffer containing 100 mM Tris pH 7.5, 5 mM DTT, 5 mM CaCl2, 2% DMSO, and 10 mM Nα-Benzoyl-L-arginine ethyl ester hydrochloride substrate (BAEE, Sigma-Aldrich #B4500) for 1 h at 37 °C. The reaction was stopped by the addition of 10 µl 5 M HClO4. For the development of color reaction product, 150 µl of developing solution (containing 0.16% w/v diacetyl monoxime, 0.0033% w/v thiosemicarbazide, and 0.16 mg/ml FeCl3 (all from Sigma-Aldrich) in 16.33% v/v H2SO4 and 11.33% v/v H3PO4 (Avantor Performance Materials Poland)) was added to the samples and incubated at 110 °C for 3 min. Absorbance of the samples was measured at 535 nm with a Hidex Sense multiplate reader (Hidex), and compared to the standard curve of L-citrulline (Sigma-Aldrich #C7629), 1 mU of PAD4 activity is defined as 1 nanomole of citrulline produced by the enzyme within 1 h of incubation at 37 °C.

Evaluation of PAD4 and PAD4 mutants activity in the presence of different concentrations of heparin/GAGs/Dp forms (GAG curves) was performed in similar manner in the presence of 0.1 mM CaCl2 with addition of heparin/GAGs/Dp concentrations 0 to 5 µM. Later on the reaction was stopped and developed as above. Activity of PAD4 in the presence of different activator concentrations presented as percent of maximal PAD4 activity in the presence of heparin.

Evaluation of PAD4 activity in the presence of different calcium concentrations (Calcium curves) with and without heparin/Dp was performed using 0 to 10 mM CaCl2 range of concentrations alone or with 1 µM Heparin/Dp. Reaction was stopped and developed as above. Activity of PAD4 in the presence of different calcium concentrations was presented as percent of maximal PAD4 activity in calcium alone.

Materials used: Heparin sodium salt from porcine intestinal mucosa, Sigma-Aldrich #H3149; GAGs and heparin oligomers all from Iduron: Dp4 (#HO04), Dp8 (#HO08), Dp12 (#HO12), Dp12 N-desulfated reN-acetylated (#reAc DSHO12), Dp16 (#HO16), Dp20 (#HO20), dermatan sulfate (#GAG-DS01), heparan sulfate (#GAG-HS01), chondroitin sulfate A (#GAG-CSA01), chondroitin sulfate C (#GAG-CSC01), chondroitin sulfate D (#GAG-CSD01). Detailed description of the BAEE activity assay is provided in *SI Appendix*.

### Histone Citrullination.

For each reaction 0.5 µg of human histone H3.1 (Sigma-Aldrich #H2292) was incubated for 2 h at 37 °C with 3 mU of PAD4 in the presence or absence of 1 µM heparin (Heparin sodium salt from porcine intestinal mucosa, Sigma-Aldrich #H3149) in 50 mM Tris pH 7.5, 5 mM DTT and 0 to 2 mM CaCl_2_. The reaction was stopped by addition of 6x concentrated sample buffer and boiling for 15 min. at 100 °C. Samples were then subsequently analyzed by western blot. Briefly, after SDS-PAGE on 10% polyacrylamide gels, proteins were transferred on PVDF membrane (Cytiva #10600023). Membranes were blocked with 5% skim milk (BioShop #SKI400) in the washing buffer (50 mM Tris pH 7.5, 150 mM NaCl, 0.05% Tween20) for 3 h at RT. Then membranes were cut based on molecular weight marker and the bottom part of the membrane (containing histone H3.1) was stained with primary rabbit antibodies anti-histone H3 Cit (Abcam #ab5103, dilution 1:2,000) overnight at 4 °C, followed by washing and staining with secondary antibodies goat anti-rabbit-HRP (Sigma-Aldrich #A0545, dilution 1:20000) for 2 h at RT. The upper part of the membrane (containing HisTag-PAD4) was stained with mouse monoclonal anti-polyHistidine−HRP antibody (Sigma-Aldrich #A7058, dilution 1:20000) for 2 h at RT. HRP signal was detected on X-ray film blue (AGFA #CP-BU NEW) with Pierce ECL substrate (Thermo Scientific #32106). For the evaluation of other GAGs influence on histone citrullination, the procedure was performed analogously but in the presence of 0.1 mM calcium chloride and respective GAG at 1 µM.

### Microscale Thermophoresis (MST).

Microscale thermophoresis was measured in 20 mM Tris pH 7.5, 300 mM NaCl, 0.05% Tween20. In the first setting, the signal was measured from FITC-Heparin (Heparin Fluorescein Conjugate, Invitrogen #H7482) at final concentration of 12.5 nM and PAD4 was added at final concentrations starting at 1 µM followed by 15 twofold dilutions. Samples were mixed, centrifuged at 16,000 g, 4 °C, 5 min and loaded into Monolith Premium Capillaries (NanoTemper #MO-K025). Samples were measured on a Monolith NT.115 (NanoTemper) instrument using nano-blue laser at 100% power. Data from initial fluorescence were used for further analysis. In the second setting in 20 mM Tris pH 7.5, 300 mM NaCl, concentration of PAD4 was kept constant at 50 nM and heparin (Heparin sodium salt from porcine intestinal mucosa, Sigma-Aldrich #H3149) was added at final concentrations starting at 5 µM followed by 15 twofold dilutions. Samples were mixed and centrifuged as before, and loaded into Monolith LabelFree capillaries (NanoTemper #MO-Z022). Measurements were done on a Monolith NT.LabelFree (NanoTemper) instrument with Labelfree laser at 80% power and medium MST setting and data from MST mode were used for analysis. In both experiments raw data were imported into MO.Affinity Analysis 2.3 software and exported for further analysis and fitting of Hill slope.

### Protein Stability Tests in the Presence of Heparin with nanoDSF.

PAD4 was used at a final concentration 0.2 mg/ml in assay buffer 50 mM Tris pH 7.5, 150 mM NaCl with varying concentrations of heparin (0 to 10 µM) (Heparin sodium salt from porcine intestinal mucosa, Sigma-Aldrich #H3149). Samples were vortexed and centrifuged at 16,000 g, 4 °C, 5 min and loaded into Prometheus High Sensitivity Capillaries (NanoTemper #PR-C006). Thermal stability was measured on Prometheus Panta (NanoTemper) in range 20 to 95 °C with 1 °C/min ramp. Data were analyzed with NanoTemper analysis software, exported, and processed using Origin software v2023 (OriginLab). The processing steps included first-order derivatization with Savitzky–Golay (SG) smoothing (polynomial order 2, points window 20), baseline subtraction using the Origin Peak Analyzer with the baseline detection ALSS method (threshold 0.1, smoothing factor 6, iterations 10), normalization of the data, and final smoothing using the SG method (order 2, points window 30). The same analysis was also performed for PAD4 with CaCl_2_ concentrations 0 to 30 mM, and PAD4 with 1 µM heparin and CaCl_2_ 0 to 30 mM.

### Susceptibility of PAD4 to Degradation by Trypsin, RgpB, Kgp, and V8 Proteases in Presence/absence of Heparin in Different Calcium Concentrations.

PAD4 (2 µg) was incubated for 1 h in 37 °C with four different types proteases: trypsin (500 nM) (Sigma-Aldrich), RgpB (Arg-specific protease from *Porphyromonas gingivalis*) (100 nM), Kgp (Lys-specific protease from *P. gingivalis*) (50 nM) and V8 (Glu-C serine protease from *Staphylococcus aureus*) (50 nM) in presence or absence of 1 µM heparin and gradient of calcium concentrations (0, 0.1, 1, 10 mM CaCl_2_). After incubation, samples were mixed with 6x concentrated sample buffer and resolved on 12% polyacrylamide gels in Schager&Jagow system, with further Coomassie BB staining. Densitometry signal was measured using Image Lab 6.1 Software (Bio-Rad). Bacterial proteolytic enzymes were a kind gift from Jan Potempa PhD, Faculty of Biochemistry, Biophysics and Biotechnology, Jagiellonian University.

### PAD4 Dimerization Mutants Particle Size Analysis.

Size of particles in solution of 0.2 mg/ml PAD4 and its mutants was analyzed in buffer consisting of 50 mM Tris, 150 nM NaCl. Protein was diluted in buffer, centrifuged at 16,000 g, 4 °C, 5 min and loaded into Prometheus High Sensitivity Capillaries (NanoTemper #PR-C006). Particle size was measured on Prometheus Panta (NanoTemper). Data were analyzed with NanoTemper analysis software and exported for visualization. Results were compared with predicted hydrodynamic radius of PAD4 monomer and dimer obtained with HullRad (39). Additionally, molecule radius was used to estimate protein molecular weight using online tool from Fidabio company (https://www.fidabio.com/molecular-weight-to-size-protein-radius-calculator).

### Heparin–PAD4 Interaction Analysis With SPR.

For immobilization on the SPR chip, heparin was first biotinylated. For biotinylation reaction 2 mg of heparin (Heparin sodium salt from porcine intestinal mucosa, Sigma-Aldrich #H3149) and 2 mg of EZ-Link Amine PEG3-biotin (Thermo Scientific #21347) were dissolved together in 200 µl HPLC grade water. The mixture was then added to 10 mg of NaCNBH_3_ (sodium cyanoborohydride, Sigma-Aldrich #156159) and incubated at 70 °C for 24 h, when an additional 10 mg of NaCNBH_3_ was added and incubated for another 24 h. Samples were concentrated to 50 µl on Pierce Concentrator PES 3 K MWCO (Thermo Scientific #88525), diluted to 500 µl with 50 mM Tris pH 7.5 and concentrated again. Dilution and concentration were repeated 6 times in total to remove leftover reagents. Finally, biotinylated heparin was brought to 200 µl. Interaction analysis by surface plasmon resonance was performed using OpenSPR two channel device (Nicoya) and Biotin-Streptavidin Sensor Kit (Nicoya #SEN-AU-100-10-STRP-KIT). Assay was performed in buffer 100 mM Tris pH 7.4, 200 mM NaCl, 0.2 mM CaCl_2_. The SPR chip was loaded into the instrument and prepared according to manufacturer instructions, biotinylated heparin was immobilized by single injection of 500 µg/ml in the assay buffer. Dilutions of PAD4 or respective mutants were prepared in the assay buffer at indicated final concentrations. Injections were performed under 20 µl/min. flow, 300 s contact time, 600 s dissociation time. After each injection of PAD4 chip was regenerated with injection of 2 M NaCl in the assay buffer, (flow 150 µl/min., contact 40 s, dissociation 270 s). Sensograms were imported and analyzed in TraceDrawer software ver. 1.9.1, data were fitted with 1:1 diffusion corrected model and bulk shift set to constant 0.1.

### CryoEM Structure Analysis.

For CryoEM analysis PAD4 (at 0.35 mg/ml final concentration) was suspended in 200 mM glycine-NaOH pH 9.3, 150 mM NaCl, with addition of CaCl_2_ when indicated. For complex formation Dp12 oligomer (Iduron #HO12) at 10 µM or Dp20 oligomer (Iduron #HO20) at 2.5 µM was added. Then, 4 µl of the sample was applied on the glow-discharged (70 s, 8 mA) copper grid (Quantifoil, copper 2/2, mesh 200, Jena Bioscience #X-103-Cu200). The sample was flash frozen using Vitrobot IV (Thermo Scientific) with 0 blot force, 4 s blot time at 4 °C and 100% humidity. Frozen sample was imaged using the Titan KRIOS microscope (Thermo Scientific) operating at 300 kV and total dose ~40 e^−^/Å^2^. Collected movies were then analyzed using cryoSPARC (40). Raw imported movies were first motion corrected (PatchMotionCorr) and CTF function was estimated (PatchCTFestimation). All micrographs were sorted in order to get rid of any junk images. Neural network–supported algorithm was employed in some analysis (Topaz) (41).

Model fitting, refinement, and validation: The previously solved crystal structure of PAD4 (PDB ID: 3APN) was manually docked using UCSF ChimeraX (42), followed by the “Dock in Map” tool in Phenix (43). Further manual model rebuilding was performed in COOT (44), followed by iterative cycles of real-space refinement in Phenix. Figures were created in UCSF ChimeraX and PyMOL (The PyMOL Molecular Graphics System, Version 2.6 Schrödinger, LLC).

Detailed description of the particle classification and data processing is provided in *SI Appendix*.

### Software and Data Analysis.

Final data visualization and analysis was done in GrapPad Prism 9.1.1 (Dotmatics). For calculating the K_D_ and Ca_0.5_ values a custom modification of specific binding with the Hill slope equation was used:

$$Y=\frac{Y_{\max}\times X^{h}}{\left(Z^{h}+X^{h}\right)}+Y_{\min},$$

where Y_max_ is maximal activity from fit, X is a concentration of either GAG or Ca^2+^, h is Hill coefficient, Y_min_ is minimum fitted activity and Z is either K_D_ or Ca_0.5_, depending on the experiment type.

Statistical analysis was done in the same software using ANOVA and Dunnett multiple comparison correction as indicated in figure description.

## Supplementary Material

Appendix 01 (PDF)

Dataset S01 (XLSX)

Dataset S02 (XLSX)

Dataset S03 (XLSX)

Dataset S04 (XLSX)

Dataset S05 (TXT)

Dataset S06 (TXT)

Dataset S07 (TXT)

## Data Availability

Cryo-EM maps were deposited in the Electron Microscopy Data Bank (EMDB) under accession numbers EMD-52412 (45), EMD-52413 (46), EMD-52414 (47), and EMD-52415 (48). Protein structures were deposited in the Protein Data Bank (PDB) under the accession numbers 9HUH (49), 9HUI (50), and 9HUJ (51). The author-accepted version of this manuscript was deposited in the preprint server Biorxiv (https://doi.org/10.1101/2024.06.17.599283) under a CC BY 4.0. international license (52).
