# Supplementary material for: Glycosaminoglycans activate peptidylarginine deiminase 4 by enhancing calcium affinity
Source: Proc Natl Acad Sci U S A. 2025 Oct 30;122(44):e2508369122. doi: 10.1073/pnas.2508369122 (PMC12595441; doi:10.1073/pnas.2508369122)
Supplement: Supplementary file 1 — Appendix 01 (PDF) [file pnas.2508369122.sapp.pdf]

PNAS

Supporting Information for
Glycosaminoglycans activate peptidylarginine deiminase 4 by enhancing calcium affinity

Grzegorz P. Bereta, Ewa Bielecka, Karolina Marzec, Łukasz Pijanowski, Artur P. Biela, Piotr Wilk, Marta Kamińska, Jakub Nowak, Elżbieta Wątor-Wilk, Przemysław Grudnik, Dominik Kowalczyk, Joanna Kozieł, Piotr Mydel, Marcin Poręba, Tomasz Kantyka

Tomasz Kantyka
Email: tomasz.kantyka@uj.edu.pl

This PDF file includes:

Supplementary Methods: Detailed method description for Recombinant PAD4 expression and purification, PAD4 activity assay with BAEE substrate and CryoEM structure analysis

Figures S1 to S15

Legends for Datasets S1 to S7

Other supporting materials for this manuscript include the following:

Datasets S1 to S7

Supplementary Methods

Recombinant PAD4 expression and purification

Full length human PAD4 sequence (GenBank NM_012387) with N-terminal affinity Histidine tag: MGHHHHHHHHHHH cloned into pET16b vector was synthesized by GenScript Biotech Corporation. Subsequent mutant variants of PAD4 were also synthesized by GenScript in the same expression vector: 1. $PAD4^{123/126/128}$ (R123S K126S K128S); 2. $PAD4^{126/128/131/134/137}$ (K126S K128S R131S K134S R137S); 3. $PAD4^{59/60/61/81/91}$ (K59S K60S K61S K81S K91S); 4. $PAD4^{8}$ (R8E); 5. $PAD4^{435}$ (Y435A); 6. $PAD^{48/435}$ (R8E Y435A). Received plasmids (10 ng) were transformed into *E. coli* BL21(DE3) expression strain using heat shock protocol: competent bacteria were mixed with DNA, incubated for 30 minutes on ice, subjected to heat shock at 42°C for 30 seconds, cooled on ice for 2 minutes. Fresh LB broth was added to bacterial suspensions and samples were incubated at 37°C with shaking for 30 minutes, then bacteria were plated on LB agar plates with 100 µg/ml ampicillin and incubated at 37°C. For expression three single colonies were inoculated into 50 ml of LB with 100 µg/ml ampicillin and incubated overnight at 37°C with shaking 180 rpm. The next day, culture was diluted 50x in 800 ml of fresh LB with 100 µg/ml ampicillin (expression was scaled up when necessary, typically 2 cultures of 800 ml were used for one purification batch) and incubated at 37°C with 180 rpm shaking until OD_{600} reached 0.6. Cultures were then incubated for 20 minutes at 4°C, expression was induced with 0.5 mM IPTG (isopropyl β-D-thiogalactopyranoside, BioShop #IPT001) and carried for 16 hours at 26°C with 180 rpm shaking. Cultures were centrifuged at 6500 g, 4°C, for 20 minutes and pelleted bacteria were collected and stored at -20°C until further purification. Cells were suspended in lysis buffer: 50 mM sodium phosphate buffer pH 7.5, 0.5 M NaCl, 0.5 mM EDTA, 10% glycerol, 0.1% Triton X-100, 10 mM imidazole on ice and sonicated for 3 minutes total pulse with pulse cycle 5 seconds on, 5 seconds off with 70% amplitude on Sonics VCX500 sonicator with 13 mm tip. Lysate was then centrifuged at 40000 g, 4°C, 40 minutes and clarified supernatant was loaded on 5 ml HisTrap FF column (Cytiva #17531901) connected to Akta Pure system (Cytiva). Column was then washed with 5 column volumes of washing buffer (50 mM sodium phosphate buffer pH 7.5, 0.5 M NaCl, 0.5 mM EDTA, 10% glycerol, 10 mM imidazole) and protein was eluted with linear gradient 0 to 100% of 500 mM imidazole in washing buffer over 6 column volumes. Fractions containing PAD4 were then subjected to buffer exchange to binding buffer: 50 mM Tris pH 7.5, 300 mM NaCl, 5% glycerol, 0.5 mM EDTA on HiPrep 26/10 Desalting column (Cytiva #17508701). PAD4 was then loaded on HiTrap Heparin HP column (Cytiva #17040601), washed with 10 column volumes of binding buffer and eluted with linear gradient 0 to 100% of 2 M NaCl in binding buffer over 15 column volumes. Protein containing fractions were pooled and subjected to size exclusion chromatography on HiLoad 16/600 Superdex 200 pg column (Cytiva #28989335) in 50 mM Tris pH 7.5, 300 mM NaCl. Finally, protein was concentrated on Amicon Ultra 15, 10000 NMWL (Millipore #UFC901024) to 3 - 5 mg/ml and stored at -80°C.

PAD4 activity assay with BAEE substrate

PAD4 activity was measured with spectrophotometric COLDER Assay (38). For general evaluation of PAD4 activity, enzyme was incubated in 50 µl reaction volume in buffer containing 100 mM Tris pH 7.5, 5 mM DTT, 5 mM $CaCl_2$, 2% DMSO and 10 mM Nα-Benzoyl-L-arginine ethyl ester hydrochloride substrate (BAEE, Sigma-Aldrich #B4500) for 1 hour at 37°C. The reaction was stopped by the addition of 10 µl 5 M $HClO_4$. For the development of color reaction product, 150 µl of developing solution (containing 0.16% w/v diacetyl monoxime, 0.0033% w/v thiosemicarbazide and 0.16 mg/ml $FeCl_3$ (all from Sigma-Aldrich) in 16.33% v/v H_2SO_4 and 11.33% v/v H_3PO_4 (Avantor Performance Materials Poland)) was added to the samples and incubated at 110°C for 3 minutes. Absorbance of the samples was measured at 535 nm with a Hidex Sense multiplate reader (Hidex), and compared to the standard curve of L-citrulline (Sigma-Aldrich #C7629), 1 mU of PAD4 activity is defined as 1 nanomole of citrulline produced by the enzyme within 1 hour of incubation at 37°C.

Evaluation of PAD4 and PAD4 mutants activity in the presence of different concentrations of heparin/GAGs/Dp forms (GAG curves) was performed in similar manner. Briefly, ~30 mU of PAD4 were incubated (37°C, 1 hour) in activity buffer described above but with 0.1 mM $CaCl_2$ with addition of heparin/GAGs/Dp concentrations 0-5 µM. Later on the reaction was stopped and developed as above. Activity of PAD4 in the presence of different activator concentrations was calculated in accordance to L-citrulline standard curve and presented as percent of maximal PAD4 activity in the presence of heparin.

Evaluation of PAD4 activity in the presence of different calcium concentrations (Calcium curves) with and without heparin/Dp was performed analogically. Briefly, ~30 mU of PAD4 was incubated in reaction buffer containing 0-10 mM $CaCl_2$ alone or with 1 µM Heparin/Dp for 1 hour at 37°C. Later on, the reaction was stopped and developed as above. Activity of PAD4 in the presence of different calcium concentrations was calculated in accordance to L-citrulline standard curve and presented as percent of maximal PAD4 activity in calcium alone.
Materials used: Heparin sodium salt from porcine intestinal mucosa, Sigma-Aldrich #H3149; GAGs and heparin oligomers all from Iduron: Dp4 (#HO04), Dp8 (#HO08), Dp12 (#HO12), Dp12 N-desulphated reN-acetylated (#reAc DSHO12), Dp16 (#HO16), Dp20 (#HO20), dermatan sulphate (#GAG-DS01), heparan sulphate (#GAG-HS01), chondroitin sulphate A (#GAG-CSA01), chondroitin sulphate C (#GAG-CSC01), chondroitin sulphate D (#GAG-CSD01).

CryoEM structure analysis

For CryoEM analysis PAD4 (at 0.35 mg/ml final concentration) was suspended in 200 mM glycine-NaOH pH 9.3, 150 mM NaCl, with addition of CaCl2 when indicated. For complex formation Dp12 oligomer (Iduron #HO12) at 10 µM or Dp20 oligomer (Iduron #HO20) at 2.5 µM was added. Then, 4 µl of the sample was applied on the glow-discharged (70 s, 8 mA) copper grid (Quantifoil, copper 2/2, mesh 200, Jena Bioscience #X-103-Cu200). The sample was flash frozen using Vitrobot IV (Thermo Scientific) with 0 blot force, 4 s blot time at 4°C and 100% humidity. Frozen sample was imaged using Titan KRIOS microscope (Thermo Scientific) operating at 300 kV and total dose ~40 e-/Å2. Collected movies were then analyzed using cryoSPARC (40). Raw imported movies were firstly motion corrected (PatchMotionCorr) and CTF function was estimated (PatchCTFestimation). All micrographs were sorted in order to get rid of any junk images.
For sample of PAD4 with Dp12, first set of particles was auto-picked using blob-picker. Particles were subjected to reference-free 2D classification to produce first 2D templates. These templates were used to search all data set to pick all similar-looking particles (Template picker). Template-picked particles were 2D classified. Particles in best-looking 2D classes were used to create initial 3D models (Ab-initio reconstruction). After heterogeneous refinement best looking 3D class was subjected to homogeneous refinement in C1 symmetry. In order to increase number of particles in the final solution, neural network-supported algorithm was employed (Topaz)(41). Homogenous refinement of the best Topaz-picked particles revealed cloud of the additional density difficult to interpret. In order to solve this part of the 3D space, all the particles were re-windowed and re-extracted in different box size. Re-windowed particles were used to train Topaz and pick a new set of particles. After Ab-initio reconstruction and refinement process final density was reconstructed.
For Dp20 without calcium, samples were prepared and imaged as described above to the stage of creation of initial 3D model (Ab-initio reconstruction). Initial model was refined (Homogenous refinement) within C1 symmetry and next, final refinement was done using Non-uniform refinement protocol.
For Dp20 with 0.1 mM CaCl2, first set of particles was auto-picked using blob-picker. Particles were subjected to reference-free 2D classification to produce first 2D templates. These templates were used to search all data set to pick all similar-looking particles (Template picker). Template-picked particles were 2D classified. Topaz was employed for particle picking, followed by 2D classification. Particles in best-looking 2D classes were used to create initial 3D models (Ab-initio reconstruction). Those models were subjected to heterogenous refinement and homogenous refinement in C1 symmetry.
For sample containing PAD4 with 10 mM CaCl2 particles were blob-picked and 2D classified to obtain templates for template-picking. As a result of template-picking best classes were selected and used in the next step to create Ab-initio volumes. Heterogenous refinement in C1 symmetry allowed to select best looking 3D volume. After first homogenous refinement, particles were unbinned and refined again. Non-uniform refinement combined with symmetry application (C2) allowed to enhance final quality of the map.
Model fitting, refinement and validation: The previously solved crystal structure of PAD4 (PDB ID: 3APN) was manually docked using UCSF ChimeraX (42), followed by the “Dock in Map” tool in Phenix (43). Further manual model rebuilding was performed in COOT (44), followed by iterative cycles of real-space refinement in Phenix. Figures were created in UCSF ChimeraX and PyMOL (The PyMOL Molecular Graphics System, Version 2.6 Schrödinger, LLC).

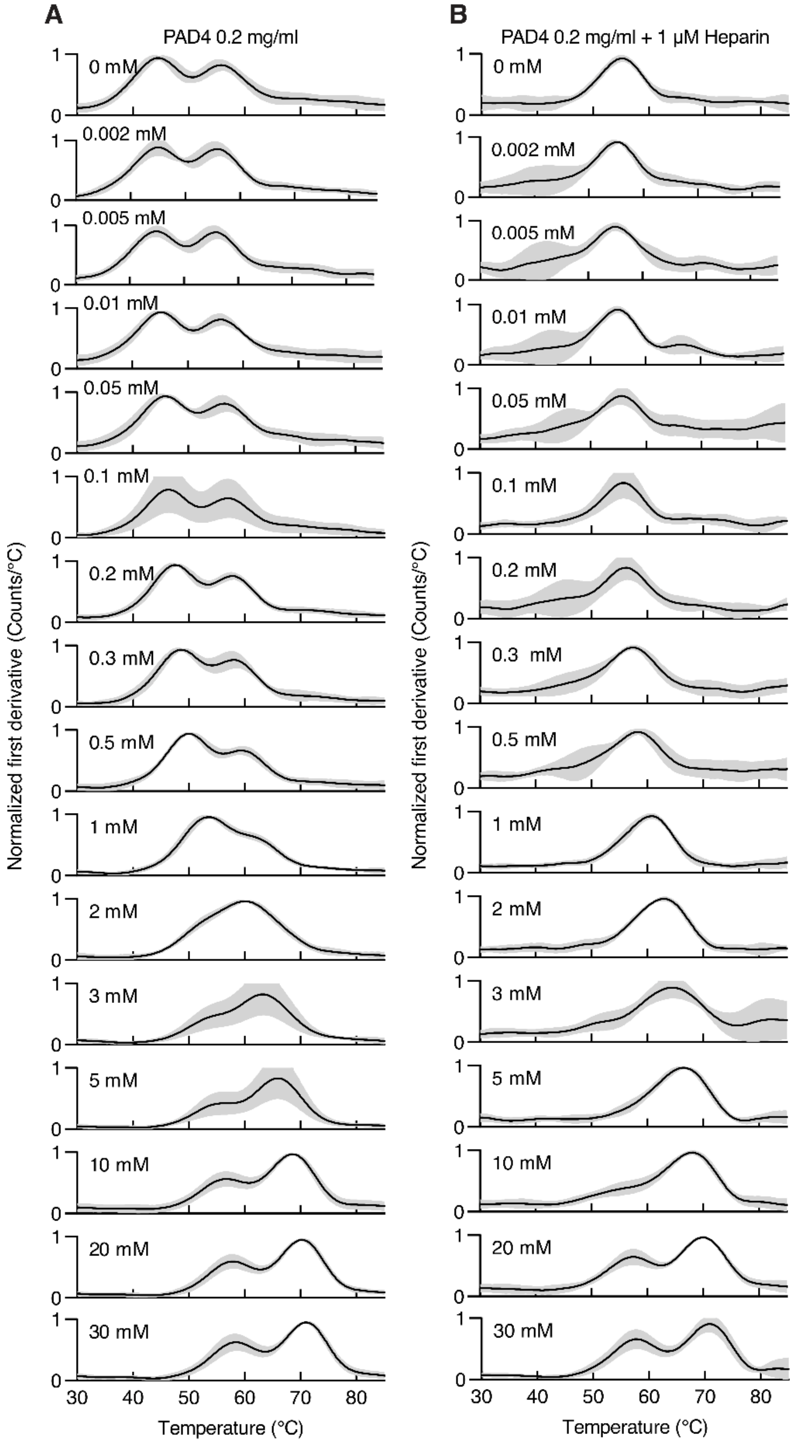

Fig. S1. Thermal unfolding of PAD4 in the presence of increasing calcium concentrations. Thermal unfolding of PAD4 (0.2 mg/ml) was measured by nanoDSF in the presence of Ca^{2+} at indicated concentrations, (**A**) without and (**B**) with 1 µM heparin. Data are presented as mean ± SD.

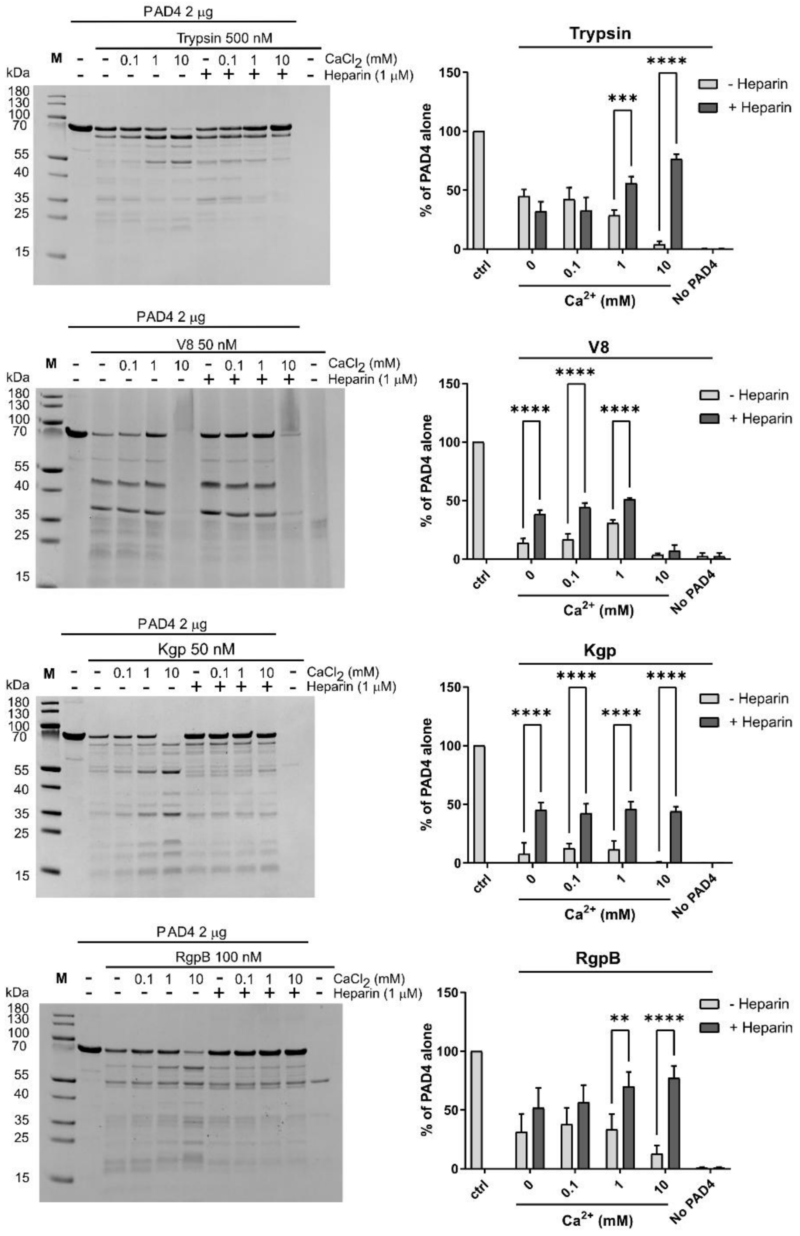

Fig. S2. Proteolytic degradation of PAD4 in the presence of calcium and heparin. Cleavage of PAD4 by 4 proteases, monitored by SDS-PAGE and Coomassie staining, M – molecular weight ladder. PAD4 was incubated with indicated proteases in the presence of increasing calcium concentrations with and without 1 µM heparin. Results were also quantified with densitometry, statistical significance of differences between sample with and without heparin at each calcium concentration was tested with two-way analysis of variance with Šídák's multiple comparisons correction **** - $p < 0.0001$, *** - $p < 0.001$, ** - $p < 0.01$ n = 3, data are presented as mean ± SD.

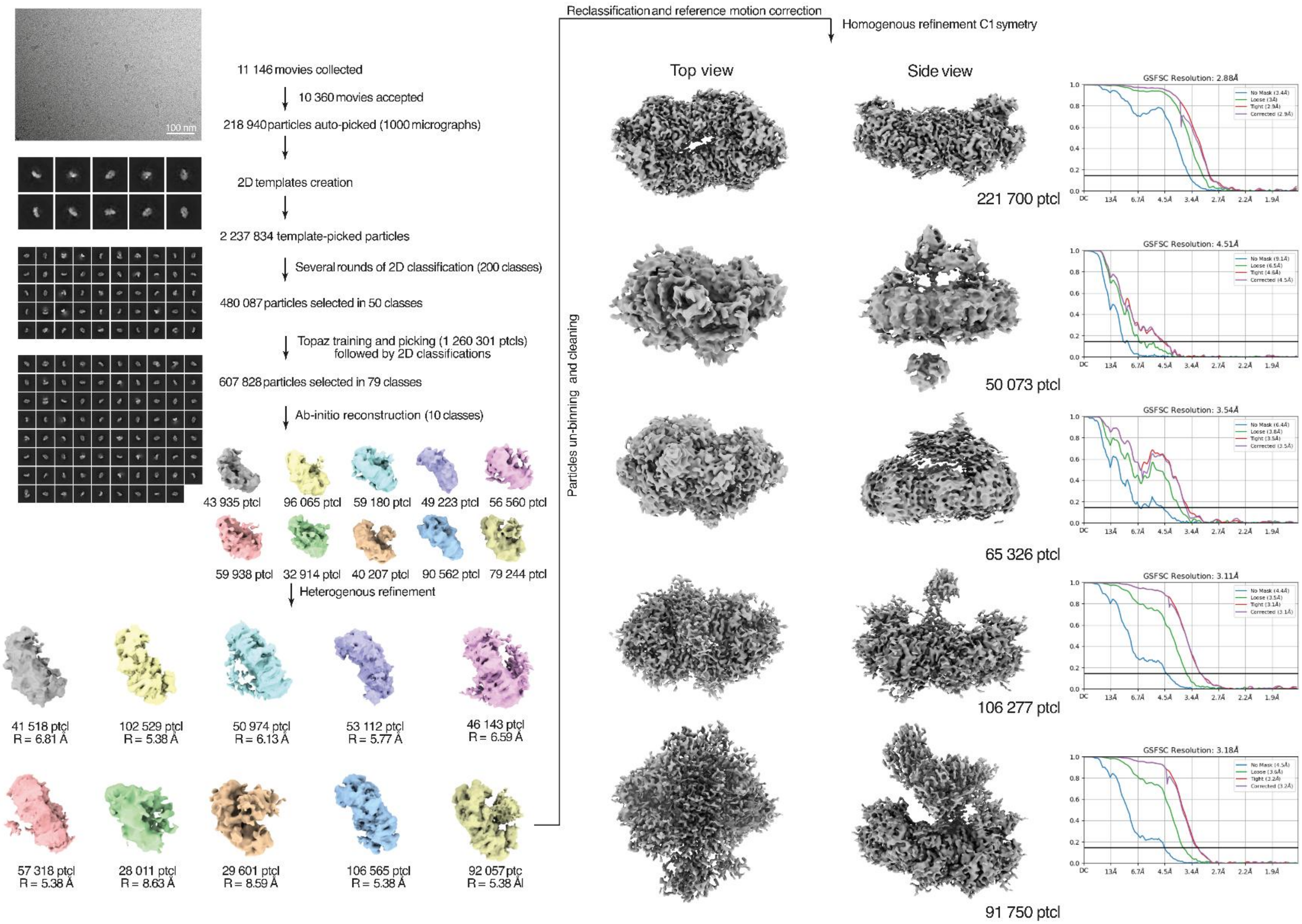

Fig. S3. CryoEM data analysis workflow of PAD4 + Dp20 heparin oligomer in 0.1 mM $CaCl_2$ dataset. Detailed presentation of each step of data analysis described in Materials and Methods, starting with example of a micrograph**,** followed by 2D class averages used for template picking, final 2D class averages used for Ab-initio reconstruction, and next analysis steps. Finally, two orthogonal views of homogenous refined volumes together with FSC correlation curves are presented.

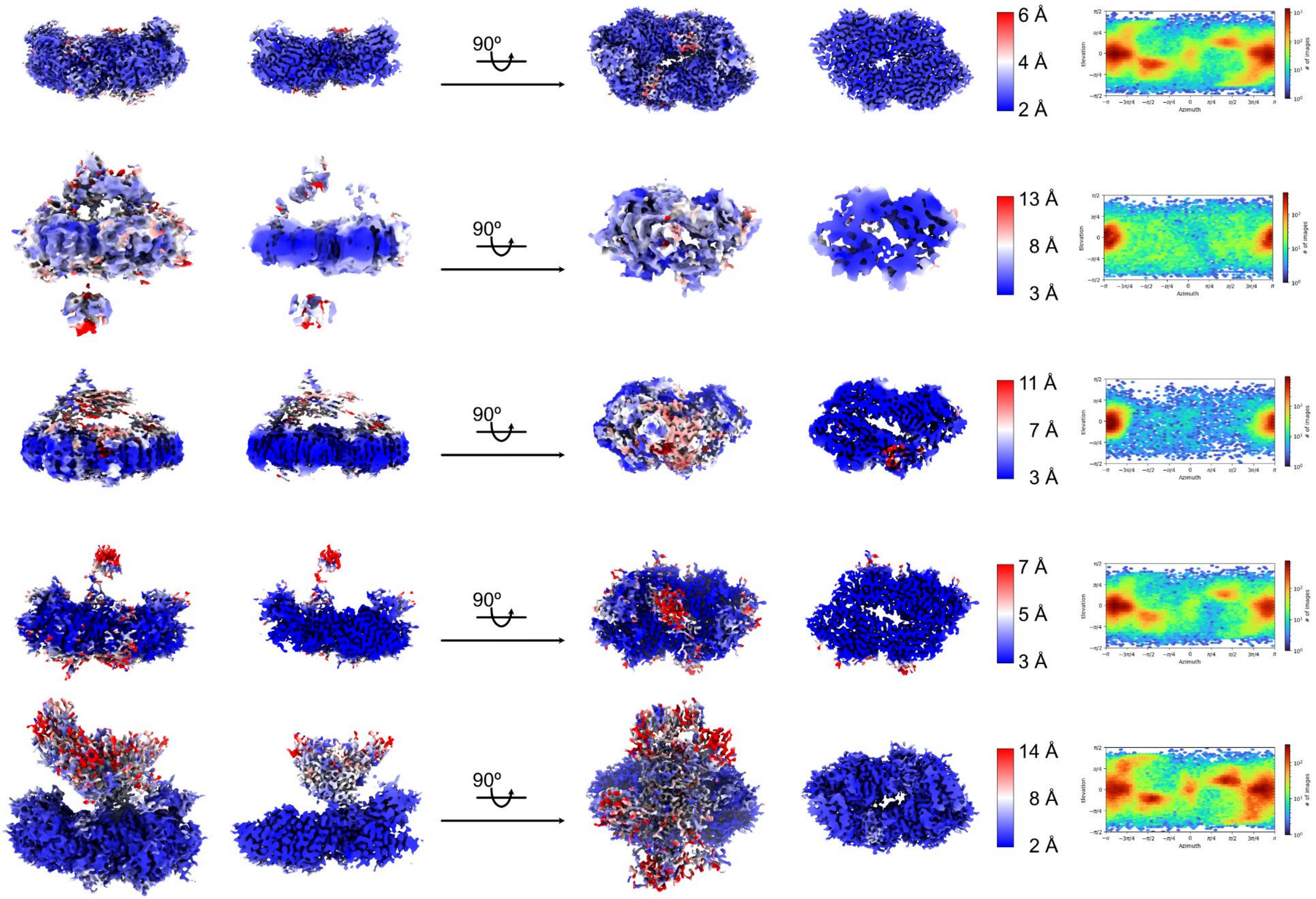

Fig. S4. CryoEM maps of PAD4 + Dp20 heparin oligomer in 0.1 mM $CaCl_2$ dataset colored by local resolution. Maps from Figure S3 were analyzed with cryoSPARC and local resolution of each refined volume is presented, additionally, directional distribution plots are provided for each volume.

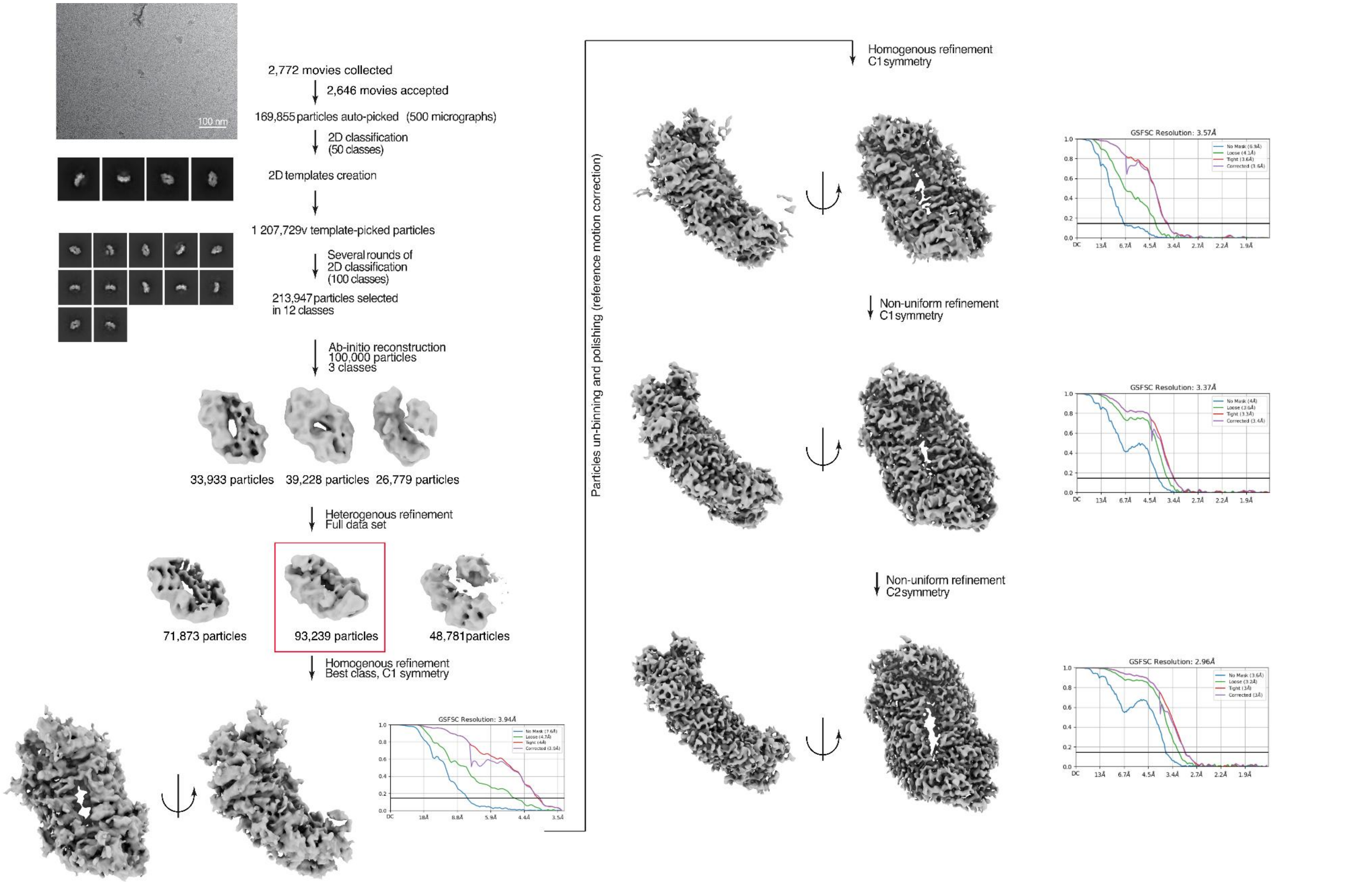

Fig. S5. CryoEM data analysis workflow of PAD4 in 10 mM $CaCl_2$ dataset. Detailed presentation of each step of data analysis described in Materials and Methods, starting with example of a micrograph, followed by 2D class averages used for template picking, final 2D class averages used for Ab-initio reconstruction, and next analysis steps. Finally, two orthogonal views of non-uniform refined volume together with FSC correlation curve are presented. Red squares along the pipeline indicate the volumes subjected for further processing.

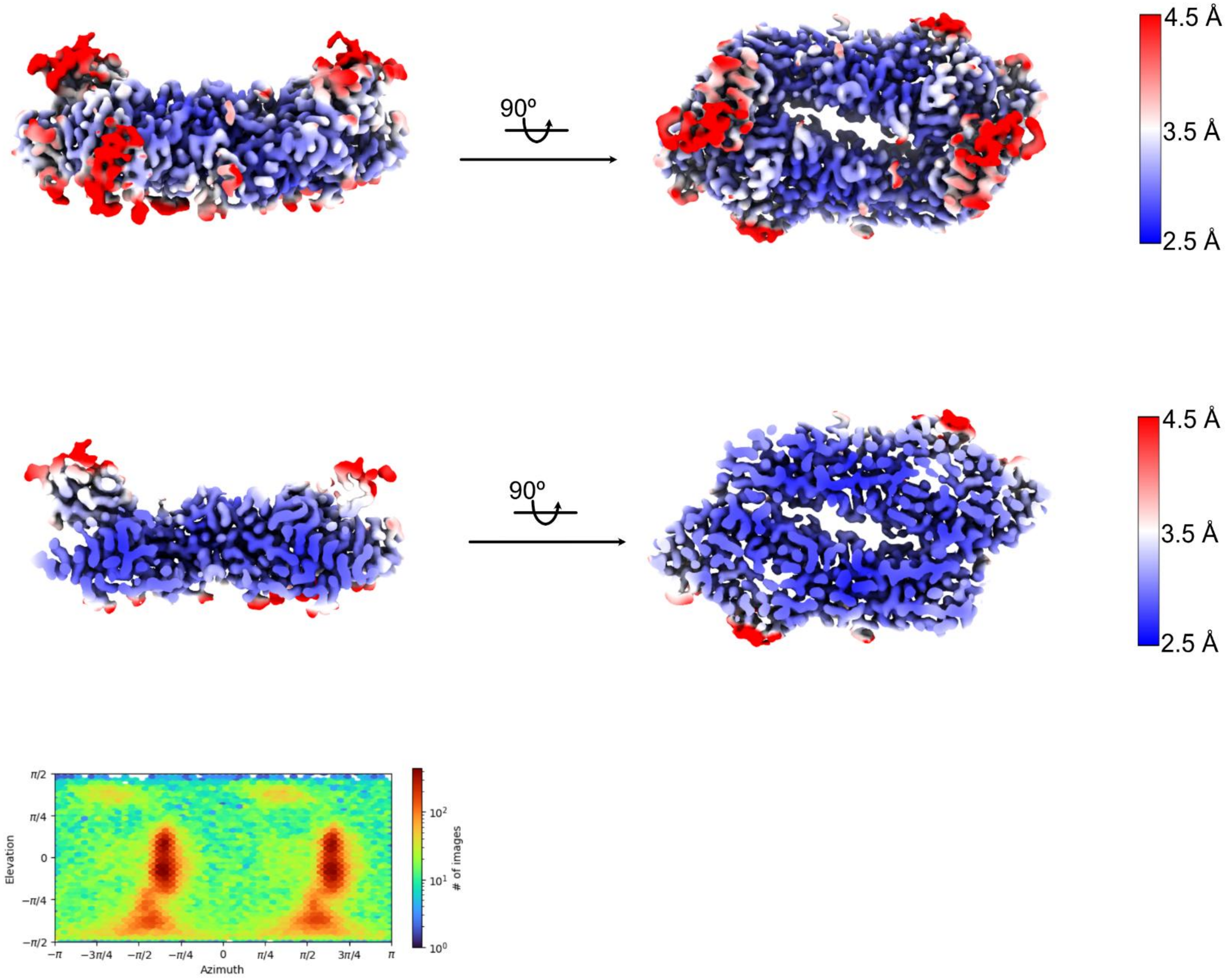

Fig. S6. CryoEM maps of PAD4 in 10 mM $CaCl_2$ dataset colored by local resolution. Maps from Figure S5 were analyzed with cryoSPARC and local resolution of each refined volume is presented, additional directional distribution plot is provided.

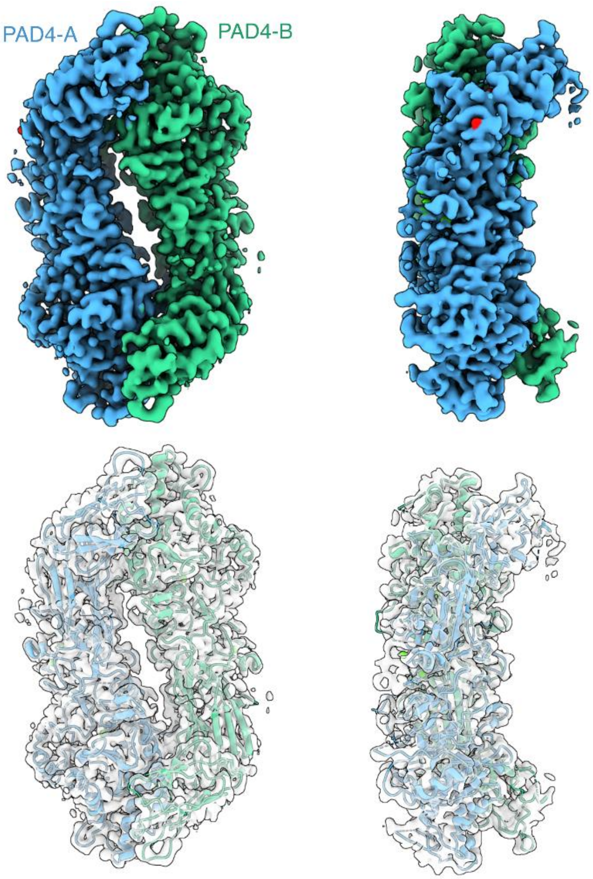

Fig. S7. CryoEM structures of PAD4 in 10 mM $CaCl_2$. CryoEM map and the corresponding model (PDB: 9HUH) in cartoon representation colored by chain with map overlaid.

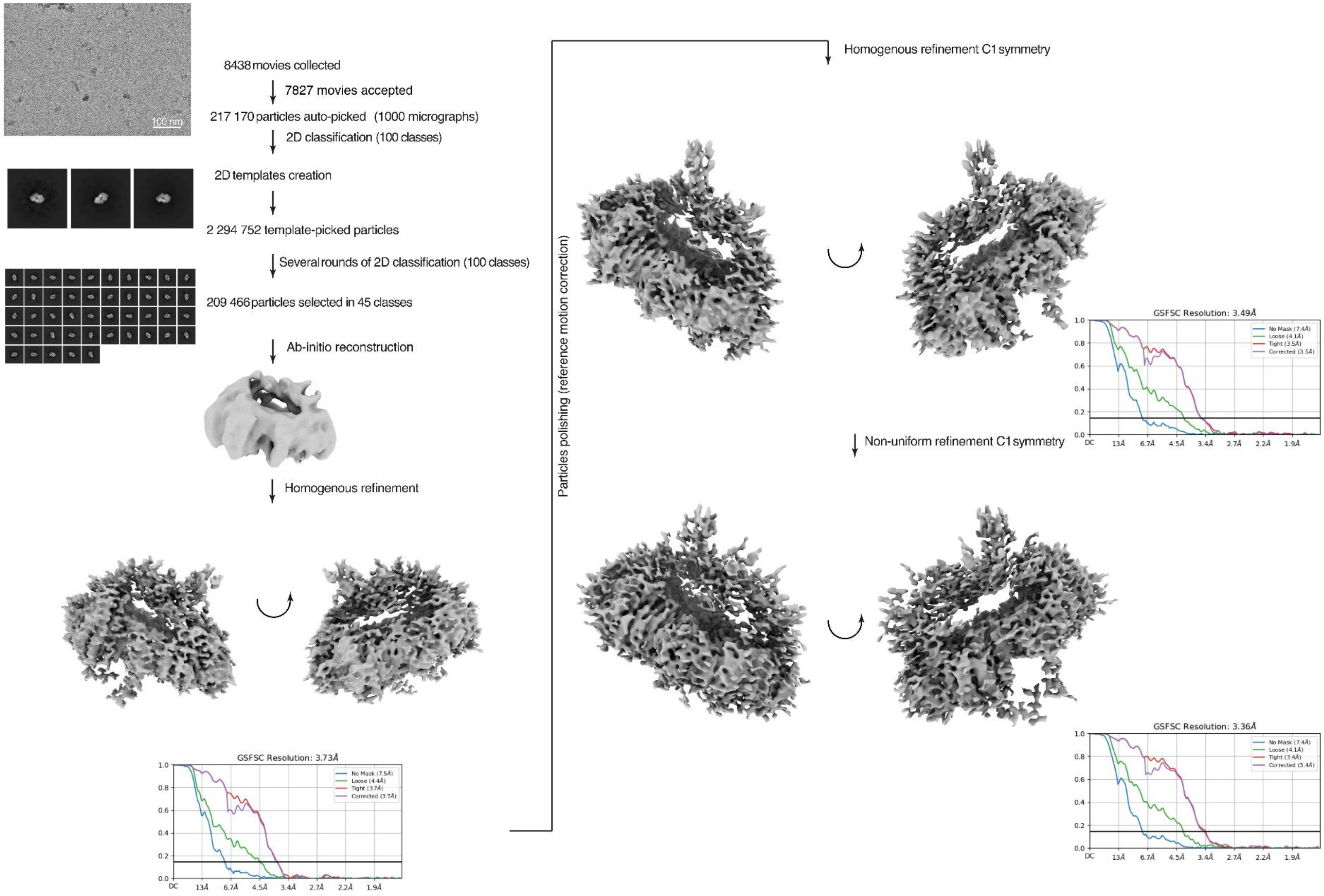

Fig. S8. CryoEM data analysis workflow of PAD4 + Dp20 heparin oligomer dataset. Detailed presentation of each step of data analysis described in Materials and Methods, starting with example of a micrograph**,** followed by 2D class averages used for template picking, final 2D class averages used for Ab-initio reconstruction, and next analysis steps. Finally, two orthogonal views of non-uniform refined volume together with FSC correlation curve are presented.

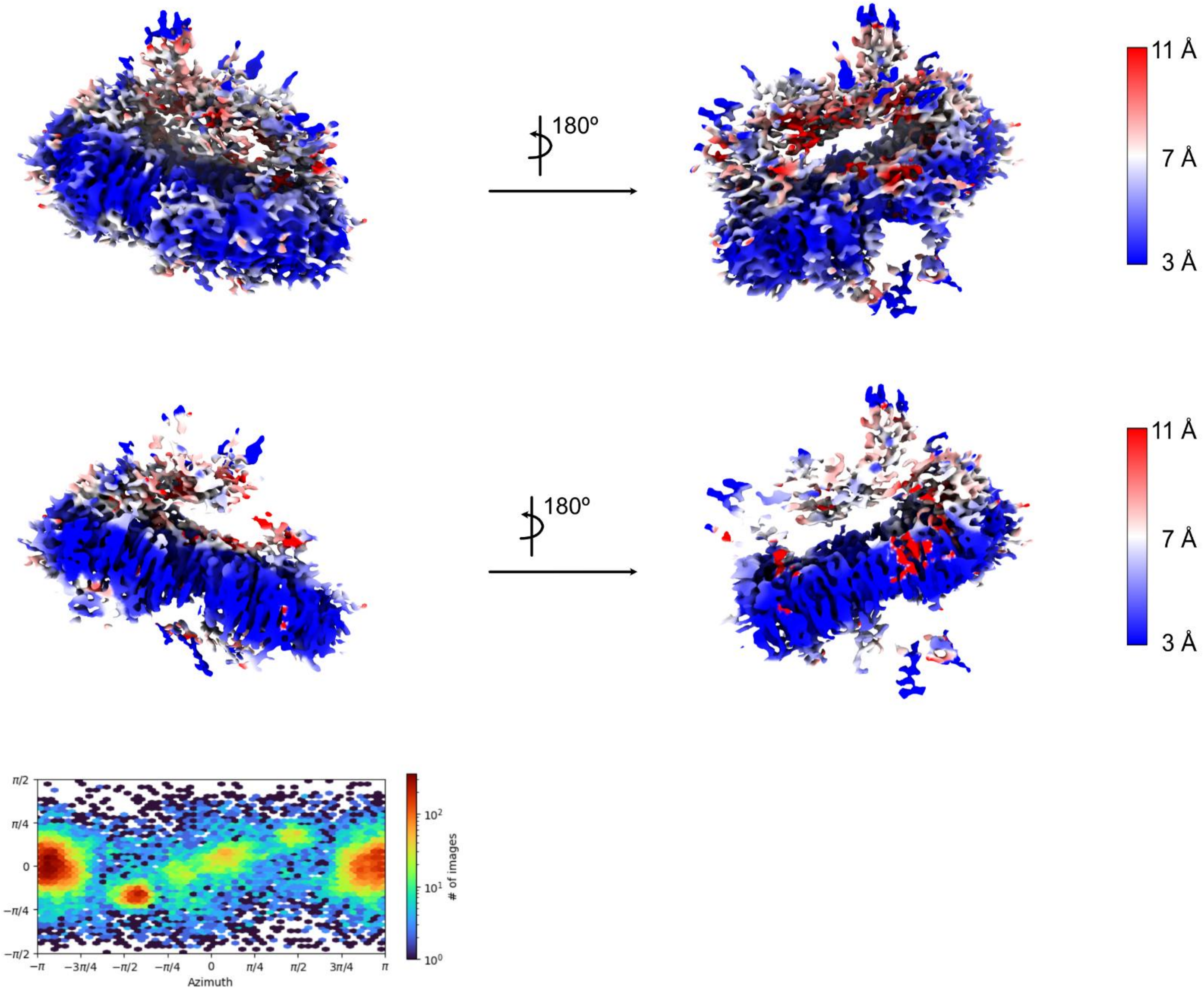

Fig. S9. CryoEM maps of PAD4 + Dp20 heparin oligomer dataset colored by local resolution. Maps from Figure S8 were analyzed with cryoSPARC and local resolution of each refined volume is presented, additional directional distribution plot is provided.

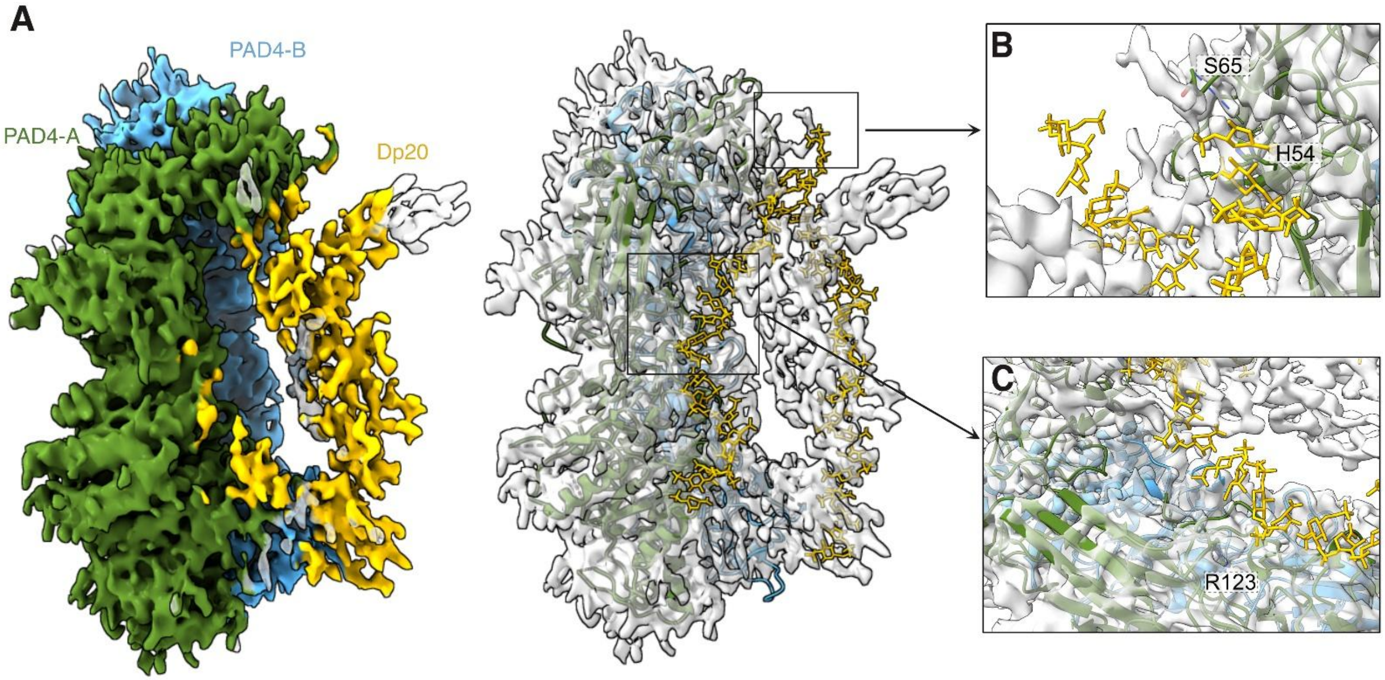

Fig. S10. CryoEM structures of PAD4 in complex with Dp20 heparin oligomer without calcium ions. (**A**) CryoEM map and the corresponding model (PDB: 9HUI) in cartoon representation colored by chain and map overlaid. The excessive density, assigned to flexible GAG chains, is colored yellow, tentative model is shown as sticks. (**B** and **C**) Close-up panels showing (**B**) region proposed to interact with Dp20 oligomer in the NLS and (**C**) in the loop around R123, flexible loops themselves were not resolved in the model. For visualization GAG chains were fitted in the constructed model.

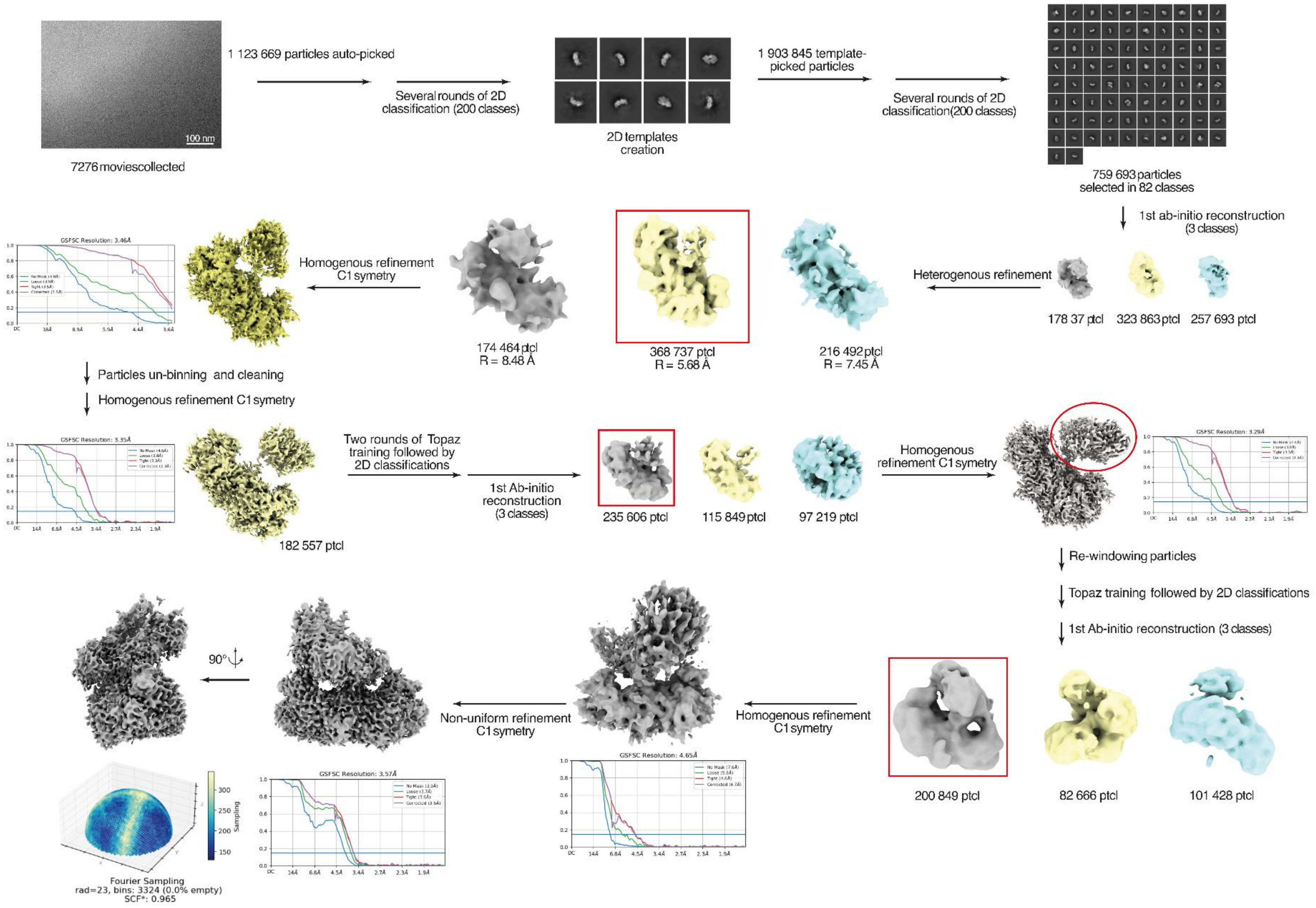

Fig. S11. CryoEM data analysis workflow of PAD4 + Dp12 heparin oligomer dataset. Detailed presentation of each step of data analysis described in Materials and Methods showing particle classes and structures obtained at each step. Starting with example of a micrograph**,** followed by 2D class averages used for template picking, final 2D class averages used for first Ab-initio reconstruction, and next analysis steps. In the homogenous refinement after Topaz – red circle points unclear and uninterpretable density. Finally, two orthogonal views of non-uniform refined volume together with directional distribution plot (bottom-left) and FSC correlation curve are presented. Red squares along the pipeline indicate the volumes subjected for further processing.

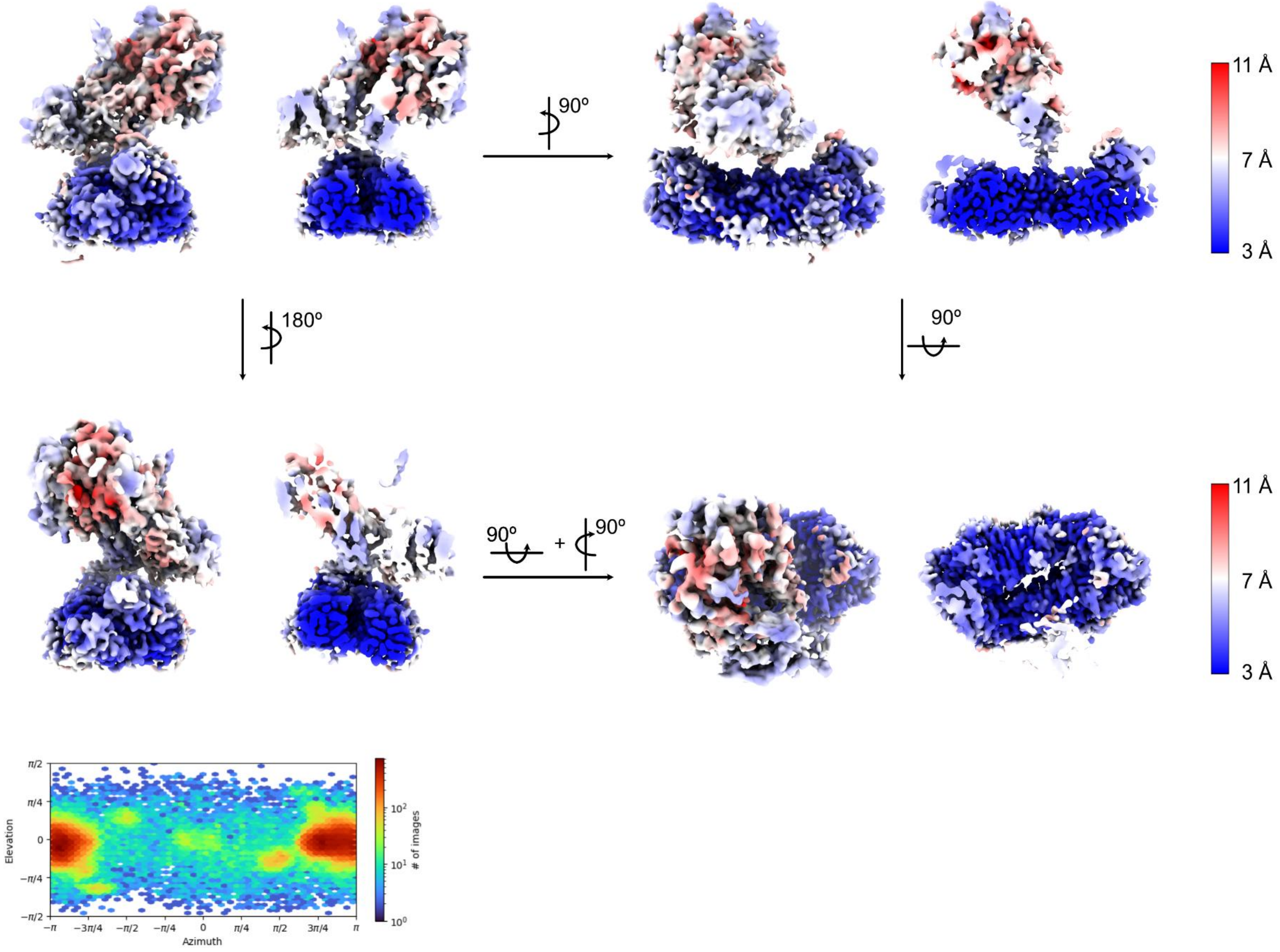

Fig. S12. CryoEM maps of PAD4 + Dp20 heparin oligomer dataset colored by local resolution. Maps from Figure S11 were analyzed with cryoSPARC and local resolution of each refined volume is presented, additional directional distribution plot is provided.

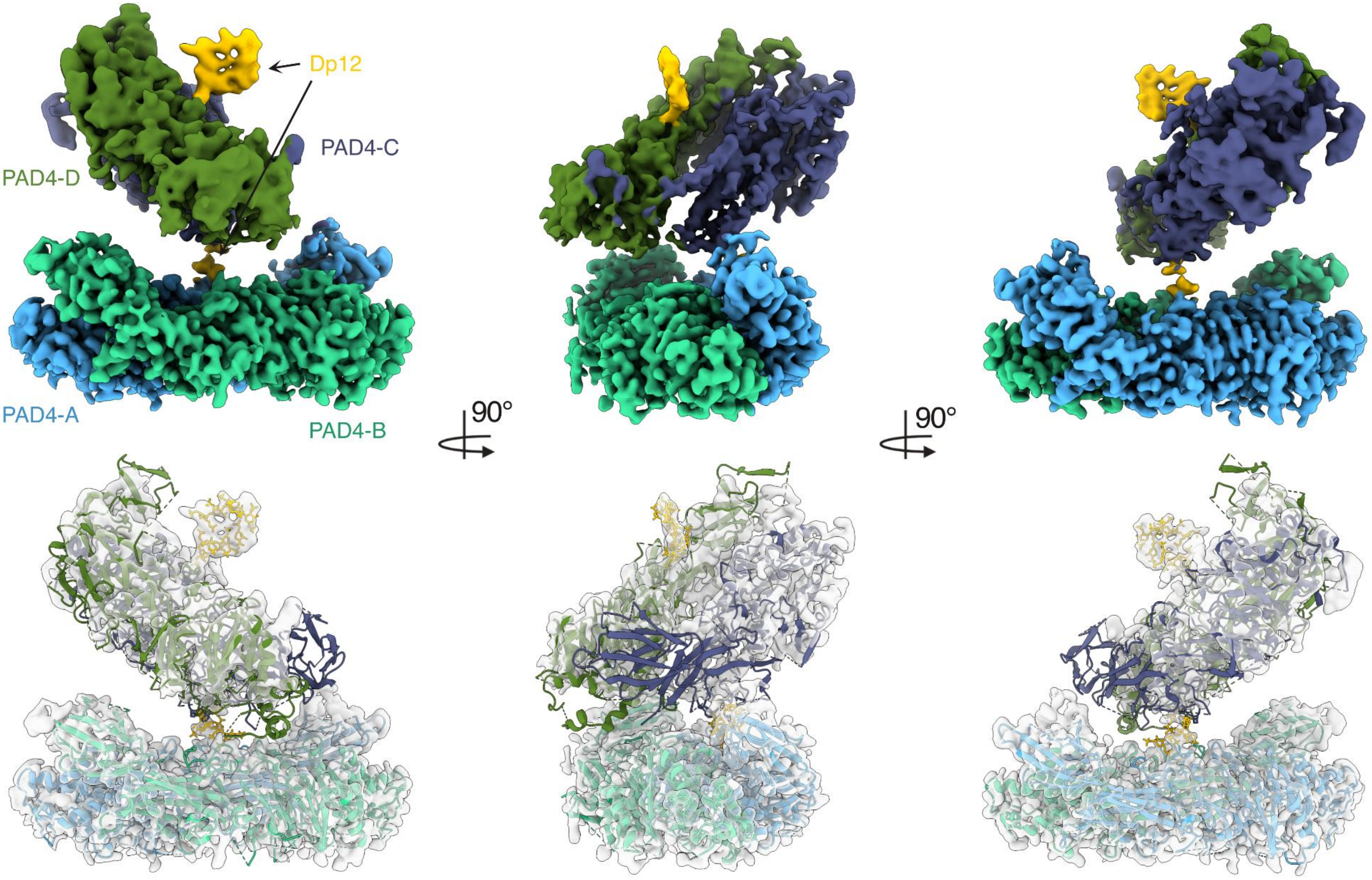

Fig. S13. Cryo EM structures of PAD4 in complex with Dp12 heparin oligomer without calcium ions. CryoEM map and the corresponding model (PDB: 9HUJ) in cartoon representation with map overlaid, colored by chain (green and blue in each PAD4 homodimer), and the excessive density assigned to flexible GAG chains is colored yellow, tentative model is shown as sticks. For visualization GAG chains were fitted in constructed model.

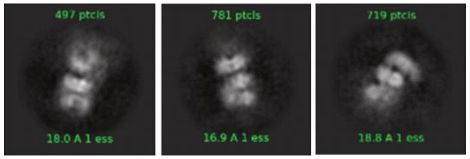

Fig. S14. CryoEM micrograph from sample of PAD4 with Dp12 heparin oligomer. 2D classes from CryoEM showing structures resembling three PAD4 dimers bounds together. Number of particles was too low to prepare 3D map of such complex.

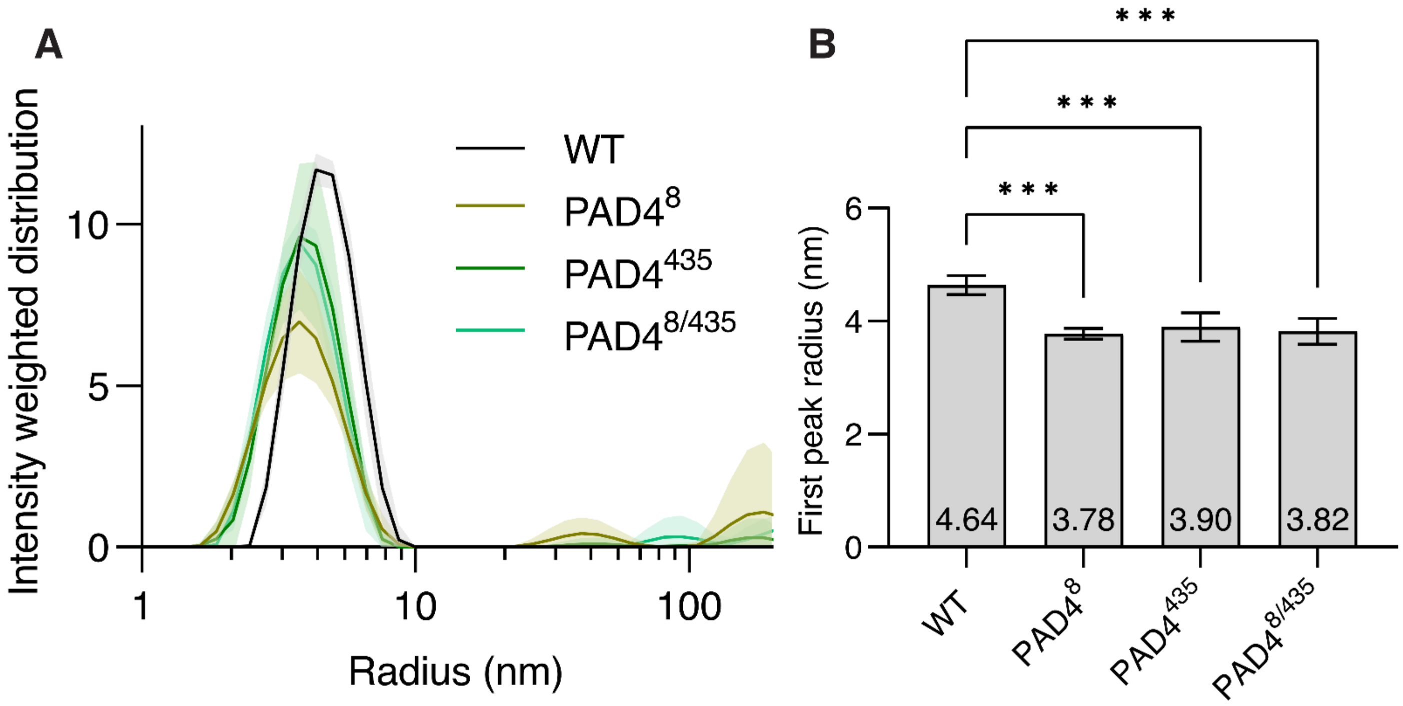

C

	PAD4 WT	$PAD4^{59/60/61/81/91}$	$PAD4^{126/128/131/134/137}$	$PAD4^{123/126/128}$	$PAD4^{8}$	$PAD4^{435}$	$PAD4^{8/435}$
Yield (mg)	9.6	6.6	10.0	5.4	8.6	15.5	9.1
Specific activity (U/mg)	158	160	193	193	112	73	44
DLS peak radius (nm)	4.64	4.77	4.53	4.49	3.78	3.90	3.82
Molecular weight from radius (kDa)	126	136	118	115	73	80	75

Fig. S15. Particle size of PAD4 dimerization mutants analyzed with DLS. Size of PAD4 particles in solution was measured in 50 mM Tris, 150 nM NaCl, 0.4 mM $CaCl_2$ for WT protein and three mutants with impaired dimerization R8E, Y435A and double mutant. (**A**) Distribution of particle sizes, mean ± SD. (**B**) Molecular radius of particles in the first peak from (A), mean ± SD, statistical significance compared to WT was tested with one-way analysis of variance with Dunnett multiple comparison correction, *** - $p < 0.001$, n = 4. (**C**) Summary of purification yields of PAD4 mutants from one batch (2x800 ml of bacterial culture), specific activity and DLS analysis of purified proteins. Expected radius for PAD4 dimer was 4.7 nm and for monomer 3.7 nm.

Dataset S1.
Table data used for preparation of figure 1.

Dataset S2.
Table data used for preparation of figure 2.

Dataset S3.
Table data used for preparation of figure 4.

Dataset S4.
Table data used for preparation of figure 5.

Dataset S5.
File containing PDB model with added GAG molecules, presented on Fig. 3.

Dataset S6.
File containing PDB model with added GAG molecules, presented on Fig. S10.

Dataset S7.
File containing PDB model with added GAG molecules, presented on Fig. S13.
